# Distinct systemic and mucosal immune responses during acute SARS-CoV-2 infection

**DOI:** 10.1038/s41590-021-01028-7

**Published:** 2021-09-01

**Authors:** Nikaïa Smith, Pedro Goncalves, Bruno Charbit, Ludivine Grzelak, Maxime Beretta, Cyril Planchais, Timothée Bruel, Vincent Rouilly, Vincent Bondet, Jérôme Hadjadj, Nader Yatim, Helene Pere, Sarah H. Merkling, Amine Ghozlane, Solen Kernéis, Frederic Rieux-Laucat, Benjamin Terrier, Olivier Schwartz, Hugo Mouquet, Darragh Duffy, James P. Di Santo

**Affiliations:** 1 Translational Immunology Lab, Institut Pasteur, Paris, France; 2 Innate Immunity Unit, Institut Pasteur, INSERM U1223, Paris, France; 3 Cytometry and Biomarkers UTechS, Institut Pasteur, Paris, France; 4 Virus and Immunity Unit, Institut Pasteur, Paris, France; 5 Sorbonne Paris Cité, Université de Paris, Paris, France; 6 Humoral Immunology Laboratory, Institut Pasteur, INSERM U1222, Paris, France; 7 DATACTIX, Paris, France; 8 Department of Internal Medicine, National Referral Center for Rare Systemic Autoimmune Diseases, Assistance Publique Hôpitaux de Paris-Centre (APHP-CUP), Université de Paris, Paris, France; 9 Imagine Institute, Laboratory of Immunogenetics of Pediatric Autoimmune Diseases, INSERM UMR 1163, Université de Paris, Paris, France; 10 Unité de Génomique Fonctionnelle des Tumeurs Solides, Centre de Recherche des Cordeliers, INSERM, Sorbonne Université, Université de Paris, Paris, France; 11 Insect-Virus Interactions Unit, Institut Pasteur, CNRS UMR2000, Paris, France; 12 Hub de Bioinformatique et Biostatistique, Institut Pasteur, Paris, France; 13 Equipe Mobile d’Infectiologie, Hôpital Cochin, AP-HP, APHP-CUP, Paris, France; 14 Université de Paris, INSERM, IAME, Paris, France; 15 Epidemiology and Antimicrobial Resistance Modeling Laboratory, Institut Pasteur, Paris, France

**Keywords:** Cytokines, Viral infection, Mucosal immunology, Adaptive immunity, Bacteria

## Abstract

Coordinated local mucosal and systemic immune responses following severe acute respiratory syndrome coronavirus 2 (SARS-CoV-2) infection either protect against coronavirus disease 2019 (COVID-19) pathologies or fail, leading to severe clinical outcomes. To understand this process, we performed an integrated analysis of SARS-CoV-2 spike-specific antibodies, cytokines, viral load and bacterial communities in paired nasopharyngeal swabs and plasma samples from a cohort of clinically distinct patients with COVID-19 during acute infection. Plasma viral load was associated with systemic inflammatory cytokines that were elevated in severe COVID-19, and also with spike-specific neutralizing antibodies. By contrast, nasopharyngeal viral load correlated with SARS-CoV-2 humoral responses but inversely with interferon responses, the latter associating with protective microbial communities. Potential pathogenic microorganisms, often implicated in secondary respiratory infections, were associated with mucosal inflammation and elevated in severe COVID-19. Our results demonstrate distinct tissue compartmentalization of SARS-CoV-2 immune responses and highlight a role for the nasopharyngeal microbiome in regulating local and systemic immunity that determines COVID-19 clinical outcomes.

## Abstract (web summary)

Mucosal surfaces of the respiratory tract are the first sites of entry and defense against SARS-CoV-2. Di Santo and colleagues perform paired analysis of the nasopharyngeal and systemic immune responses of SARS-CoV-2-infected patients and demonstrate distinct compartmentalization of immunity and shifts in the microbiome.

## Main

While SARS-CoV-2 infection is responsible for COVID-19, the regulatory mechanisms underlying disease pathophysiology remain enigmatic. Clinical manifestations following SARS-CoV-2 infection are highly variable, ranging from asymptomatic or mild symptoms to severe pneumonia that can progress to acute respiratory distress syndrome^1^. It is still unclear whether disease progression is related to the viral infection itself, to the host immune response, to host comorbidities or to a combination of these different factors^2^. Biomarkers to distinguish disease progression in COVID-19 include interleukin (IL)-6, C-reactive protein (CRP), D-dimers and lactic dehydrogenase (LDH), yet our understanding of their role in disease pathophysiology remains limited^2,3^.

Analysis of immune responses in patients with COVID-19 showed that SARS-CoV-2 suppresses activation of the innate immune system, including dendritic cells^4^ and dampens antiviral type I and type III interferon responses^5^, in parallel to an excessive proinflammatory macrophage activation^6^. Despite overall peripheral lymphopenia, patients with COVID-19 mount efficient SARS-CoV-2-specific memory T and B cell responses^7^. In particular, patients with COVID-19 show increased numbers of plasma cells and generate specific neutralizing antibodies to the SARS-CoV-2 spike protein. Virus-specific T cell responses in the blood increase with disease severity suggesting that a deficiency in adaptive immunity is not causal during early stages^8^.

One severe clinical manifestation in patients with COVID-19 is an extensive systemic immune reaction triggered by the excessive production of inflammatory mediators such as monocyte chemoattractant protein-1 (MCP-1/CCL2), macrophage inflammatory protein-1 alpha (MIP-1α/CCL3), IL-6, tumor necrosis factor (TNF) and IL-10 (ref. ^9^). SARS-CoV-2-associated hyperinflammation can promote a pathological hypercoagulable state with increased mortality for patients with COVID-19 (ref. ^6^). The systemic hyperinflammation correlates with peripheral SARS-CoV-2 RNA loads suggesting that it represents a form of ‘viral’ sepsis^10^. Still, the exact mechanism underlying this phenomenon remains to be determined.

Upon initial exposure, SARS-CoV-2 is thought to infect human angiotensin-converting enzyme 2 (hACE2)-expressing epithelial cells in the upper respiratory tract^11^. At this stage, early defense mechanisms likely limit viral replication in most individuals and prevent further disease progression. These may include physiochemical barriers (mucus and metabolites), as well as innate immune defense proteins (cytokines and interferons) that are constitutively produced or induced upon infection. Adaptive immune mechanisms, including secretory IgA, play a critical role in barrier function at mucosal sites. In the context of SARS-CoV-2 infection, several studies have documented the presence of virus-specific IgG and IgA in blood, saliva and nasopharyngeal samples of patients with COVID-19 (refs. ^12–14^). Still, how local and systemic immunity following SARS-CoV-2 infection is established and the factors that regulate this process are poorly understood.

Here we applied an integrated systems approach to identify the factors that regulate local and systemic immunity to SARS-CoV-2 using a cohort of patients with COVID-19 with varying clinical severity. Our results reveal distinct responses between nasopharyngeal and systemic immunity, with a strong impact on the nasopharyngeal cytokine response and microbiome in severe COVID-19. These results suggest new strategies for the management of patients infected with SARS-CoV-2.

## Results

### Systemic and mucosal antibody responses in patients with COVID-19

**Fig. 1:**
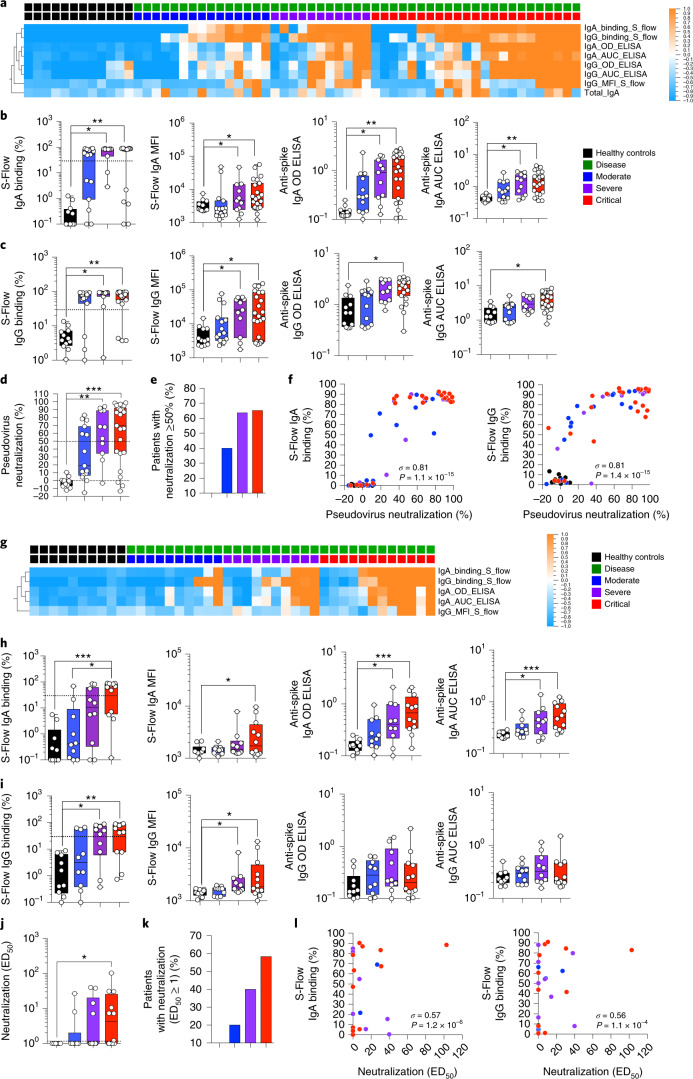
Systemic and mucosal antibody responses in patients with COVID-19. **a**–**i**, Antibodies were measured in the plasma (**a**–**c**) of healthy controls (*n* = 12 donors) and patients with mild-to-moderate (*n* = 15), severe (*n* = 11) and critical (*n* = 23) disease or in the nasopharyngeal compartment (**g**–**i**) of healthy controls (*n* = 10 donors) and patients with mild-to-moderate (*n* = 10), severe (*n* = 10) and critical (*n* = 12 patients) disease using an ELISA-based approach with soluble CoV-2 spike protein (OD and AUC ‘ELISA’) and the ‘S-Flow’ FACS-based approach using cell lines stably expressing surface CoV-2 spike (‘S-Flow’). Heat map of statistically different (*P* < 0.05) antibody responses between healthy controls and patients with COVID-19 (moderate, severe and critical) in plasma (**a**) and nasopharyngeal compartment (**g**). In **b** and **c**, and **h** and **i**, individual antibodies responses by patient severity are shown. **d**–**f**, Pseudovirus neutralization in plasma samples from healthy controls (*n* = 12) and patients with mild-to-moderate (*n* = 15), severe (*n* = 11) and critical (*n* = 23) COVID-19. **d**, The percentage of pseudovirus neutralization against the SARS-CoV-2 spike protein was measured by analyzing luciferase-expressing pseudotypes. **e**, The percentage of patients with pseudotype neutralization above 50%. **f**, Correlation plots between the pseudotype neutralization (%) and presence of anti-spike IgA or IgG as measured by S-Flow. **j**–**l**, SARS-CoV-2 neutralization by nasopharyngeal samples from healthy controls (*n* = 10) and patients with mild-to-moderate (*n* = 10), severe (*n* = 10) and critical (*n* = 12) COVID-19. **j**, SARS-CoV-2 virus neutralization was measured using an S-Fuse assay that reads out productive infection by SARS-CoV-2. Neutralizing activity of each sample was expressed as the 50% effective dose (ED_50_). **k**, Graph represents the percentage of patients with virus neutralization. **l**, Correlation plots between the neutralization (ED_50_) and presence of anti-Spike IgA or IgG as measured by S-Flow. In **a** and **g**, *P* values were determined with a two-tailed Mann–Whitney test between healthy and infected individuals. In **b**–**d** and **h**–**j**, box-and-whisker plots show the minimum, maximum, interquartile range and the median. *P* values were determined with the one-sided Kruskal–Wallis test followed by Dunn’s post hoc test for multiple comparisons with Geisser–Greenhouse correction. In **f** and **l**, *σ* represents the Spearman coefficient; *P < 0.05; **P < 0.01; ***P < 0.001. In **a** and **g**, *z*-score scale is indicated, with upregulation shown in orange and downregulation shown in blue. AUC, area under the curve; MFI, mean fluorescence intensity; OD, optical density.

**Extended Data Fig. 1:**
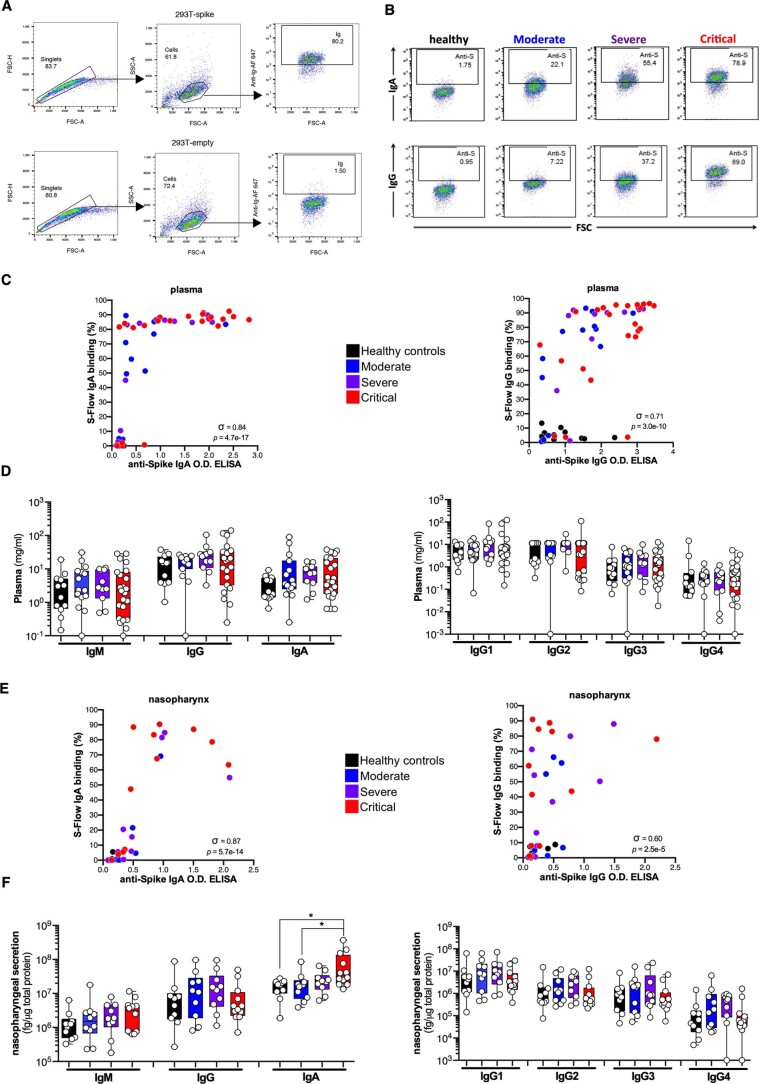
Systemic and mucosal antibody responses in patients with COVID-19. (**a, b**) Gating strategy and representative results for the S-Flow assay. (**c**) Correlation plots between the anti-Spike IgA or IgG OD ELISA and the S-Flow anti-Spike IgA or IgG binding (%) in plasma. (**d**) Total IgM, IgG and IgA and IgG1/2/3/4 were measured in plasma using a bead-based multiplexed immunoassay system Luminex. (**e**) Correlation plots between the anti-Spike IgA or IgG OD ELISA and the S-Flow anti-Spike IgA or IgG binding (%) in the nasopharyngeal compartment. (**f**) Total IgM, IgG and IgA and IgG1/2/3/4 were measured in nasopharyngeal compartment. (**a-f**) Antibodies were measured in the plasma of healthy controls (n = 12 donors), mild to moderate (n = 15 patients), severe (n = 11 patients) and critical (n = 23 patients) or in the nasopharyngeal compartment of healthy controls (n = 10 donors), mild to moderate (n = 10 patients), severe (n = 10 patients) and critical (n = 12 patients). Nasopharyngeal IgA (Critical vs Healthy, p = 2.9 × 10-2; Critical vs Moderate, p = 4.0 × 10-2). In (**c**) and (**e**), σ represents Spearman coefficient and p the p value. In (**d**) and (**f**), box-and-whisker plots showing the minimum, maximum, interquartile range and the median. P values were determined with one-sided Kruskal-Wallis test followed by with Dunn’s post-test for multiple group comparisons with Geisser-Greenhouse correction; For all panels: *P < 0.05; **P < 0.01; ***P < 0.001.

While a substantial literature exists concerning systemic humoral and cellular immune responses during SARS-CoV-2 infection^4–9,15^, we have scant knowledge concerning how mucosal immunity is established and coordinated in patients with COVID-19. To better understand these related processes, we compared immune responses in paired plasma and nasopharyngeal samples from acutely hospitalized patients with COVID-19 and healthy controls. The COVID-19 patient cohort consisted of PCR-confirmed disease at 8–12 d after symptom onset with distinct clinical classification (indicated here as moderate, severe and critical^5^; see Methods for cohort details) before treatment intervention as well as non-COVID-19 controls. We first assessed SARS-CoV-2-specific antibody responses using two complementary and sensitive assays to measure spike-specific IgG and IgA: an ELISA-based approach using soluble trimeric CoV-2 spike protein and the ‘S-flow’ fluorescence-activated cell sorting (FACS)-based approach using a cell line stably expressing surface SARS-CoV-2 spike protein (Methods, ref. ^16^ and Extended Data Fig. 1a,b). In line with previous reports^4,17^, we detected spike-specific IgG and IgA antibodies in plasma of patients with COVID-19 (*n* = 49) but not in healthy controls with an increasing frequency and intensity dependent on disease severity (Fig. 1a–c and Extended Data Fig. 1c). The neutralization activity of plasma samples against SARS-CoV-2 was clearly induced following SARS-CoV-2 infection, present in 28 of 40 seropositive individuals (70%) and increased with clinical severity (Fig. 1d,e). Moreover, neutralization intensity was highly correlated with frequency of spike-specific IgG and IgA (Fig. 1f). We did not find significant differences in total plasma IgM, IgG and IgA levels or in IgG subclass levels between healthy individuals and patients with COVID-19 (Extended Data Fig. 1d).

We applied the same antibody assays to nasopharyngeal samples (*n* = 42) collected at the same time as the plasma from a majority of patients in this COVID-19 cohort. As with plasma, we found significantly increased frequency and intensity of spike-specific IgG and IgA responses in nasopharyngeal secretions as disease severity increased (Fig. 1g–i and Extended Data Fig. 1e). Using a recently described S-Fuse assay^18^, we detected neutralizing antibodies in 13 of 17 ‘naso-positive’ patients with COVID-19 (76%) that increased with disease severity and strongly correlated with the presence of spike-specific IgA and IgG (Fig. 1j–l). Interestingly, nasopharyngeal total IgA (but not total IgM or IgG or IgG subclass) levels were significantly elevated in patients with critical COVID-19 (Extended Data Fig. 1f). These results confirm and extend previous reports of robust local and systemic humoral responses against the SARS-CoV-2 spike protein in acute COVID-19 infection^12^.

### Heterogeneous antibody responses in patients with COVID-19

**Fig. 2:**
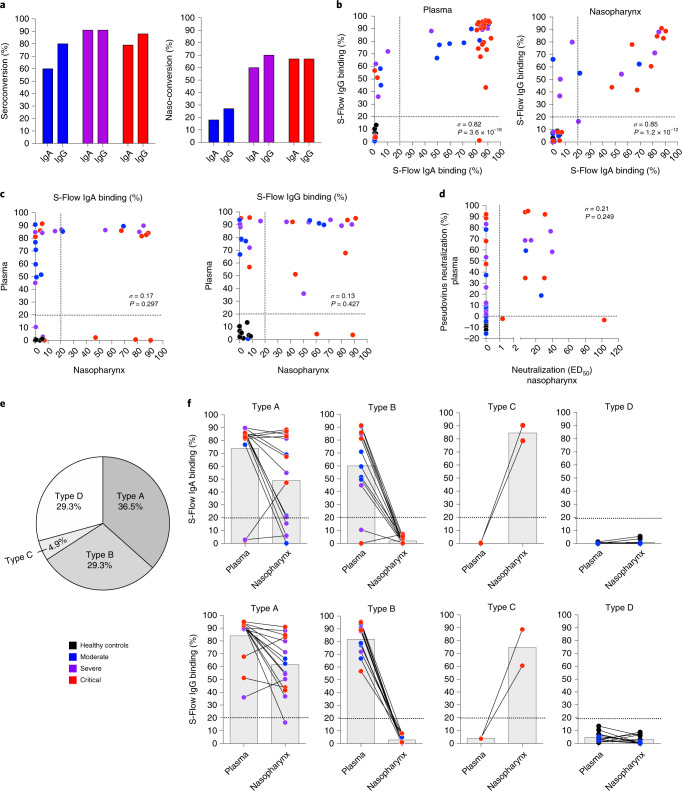
Heterogeneous systemic and mucosal SARS-CoV-2 antibody responses. **a**–**f**, IgA and IgG were assessed by S-Flow using cell lines stably expressing surface CoV-2 spike protein in plasma of healthy controls (*n* = 12) and patients with mild-to-moderate (*n* = 15), severe (*n* = 11) and critical (*n* = 23) COVID-19 or in the nasopharyngeal compartment of healthy controls (*n* = 10) and patients with mild-to-moderate (*n* = 10), severe (*n* = 10) and critical (*n* = 12) COVID-19. **a**, The percentage of IgA and IgG seroconversion in plasma and ‘naso-conversion’ (percentage of positive samples from the nasopharynx) versus disease severity. **b**, Correlation plots between the percentage of S-Flow anti-spike IgA binding and the percentage of S-Flow anti-Spike IgG binding in plasma (*n* = 61) and in nasopharynx (*n* = 42). **c**, Correlation plots between plasma and nasopharynx anti-spike antibody responses (*n* = 41). **d**, Correlation plot between the percentage of plasma pseudovirus neutralization and nasopharyngeal virus neutralization (ED_50_; *n* = 41). **e**, Representation of antibody conversion among the patients; type A represents naso-positive and seropositive patients, type B represents naso-negative and seropositive patients, type C represents naso-positive and seronegative patients and type D represents naso-negative and seronegative patients. **f**, Representation of anti-spike responses in the different compartment for each patient type. In **b**, **c** and **d**, *σ* represents the Spearman coefficient.

**Extended Data Fig. 2:**
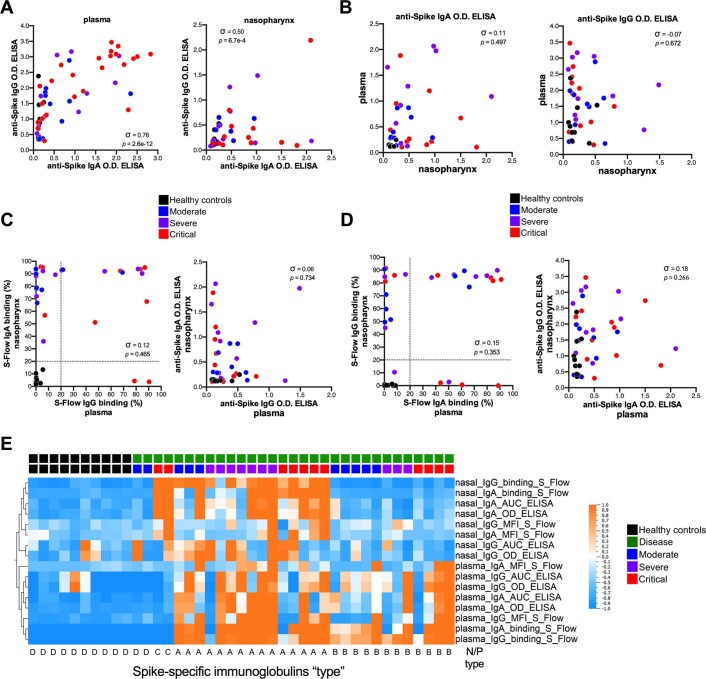
Compartmentalized spike-specific antibody responses in COVID-19. (**a**) Correlation plots between the anti-Spike IgA OD ELISA and anti-Spike IgG OD ELISA in plasma and nasopharynx. (**b**) Correlation plots between the anti-Spike IgA or IgG D.O. ELISA in plasma versus nasopharynx. (**c**) Correlation plots between plasma S-Flow anti-Spike IgG binding (%) versus anti-Spike IgG OD ELISA and nasopharyngeal S-Flow anti-Spike IgA binding (%) versus anti-Spike IgA OD ELISA. (**d**) Correlation plots between plasma S-Flow anti-Spike IgA binding (%) versus anti-Spike IgA OD ELISA and nasopharyngeal S-Flow anti-Spike IgG binding (%) versus anti-Spike IgG OD ELISA. (**e**) Heatmap representation of all antibodies measured in plasma and nasopharyngeal compartment. (**a-d**) Antibodies were measured in the plasma of healthy controls (n = 12 donors), mild to moderate (n = 15 patients), severe (n = 11 patients) and critical (n = 23 patients) or in the nasopharyngeal compartment of healthy controls (n = 10 donors), mild to moderate (n = 10 patients), severe (n = 10 patients) and critical (n = 12 patients). In (**a, b, c, d)**, σ represents Spearman coefficient and p the p value. For all panels: *P < 0.05; **P < 0.01; ***P < 0.001.

We next explored the relationship between local mucosal and systemic spike-specific antibody production during acute SARS-CoV-2 infection. This analysis confirmed previous reports^12^ but also revealed unexpected patterns of anti-SARS-CoV-2 humoral immunity. First, the majority (88%) of patients with COVID-19 seroconverted with spike-specific antibodies in their blood, which appeared to be independent of disease severity (Fig. 2a). Both spike-specific IgG and IgA were present in the majority of these seropositive individuals with COVID-19 (Fig. 2b and Extended Data Fig. 2a). Second, overall ‘naso-conversion’ (presence of spike-specific IgG or IgA in nasopharyngeal secretions) was substantially less frequent than that observed for seroconversion (Fig. 2a). Nevertheless, in these ‘naso-converters’, spike-specific IgG and IgA were still largely co-detected (Fig. 2b and Extended Data Fig. 2a). Finally, a small fraction of patients with COVID-19 (12%; 6/49) did not show IgG or IgA seroconversion (Fig. 2a), despite having been exposed to SARS-CoV-2 (all patient diagnoses of COVID-19 were confirmed by PCR). Together, these results suggest a complex patient-specific control of local mucosal and systemic antibody responses at this early time point (days 8–12) following SARS-CoV-2 infection.

We next compared systemic and local mucosal spike-specific IgG and IgA responses in individuals with COVID-19. Surprisingly, systemic (plasma) and local (nasopharynx) spike-specific antibody responses within individuals were poorly correlated (Fig. 2c). This was apparent when comparing spike-specific IgG or IgA responses in plasma versus nasopharynx or following a cross comparison of IgG with IgA responses (Extended Data Fig. 2b–d). Neutralization activity was likewise poorly correlated between nasopharyngeal and blood samples (Fig. 2d). These results suggest independent regulation of mucosal and systemic immune responses to SARS-CoV-2.

We next subclassified patients with COVID-19 and controls based on the presence or absence of spike-specific IgG and/or IgA in the plasma (P) or nasopharynx (N) as type A (PN^+^; 29.3%), type B (P^+^; 36.5%), type C (N^+^; 4.9%) or type D (PN^−^; 29.3%) responders (Fig. 2e,f and Extended Data Fig. 2e). As expected, all controls were type D (seronegative/naso-negative), but two patients with moderate COVID-19 were seronegative and naso-negative at this time point (Fig. 1a,g). Interestingly, two patients with critical COVID-19 showed an absence of spike-specific antibodies in the plasma but strong spike-specific IgG and IgA responses in the nasopharynx (Fig. 2f and Extended Data Fig. 2e), thus identifying these patients as type C responders. The remaining patients with COVID-19 were split between type A and type B responders and were not enriched for any particular disease severity (Extended Data Fig. 2e). Taken together, these results demonstrate heterogeneous SARS-CoV-2 antibody responses within systemic and local mucosal sites (at 8–12 d after symptom onset), suggesting tissue-dependent regulation.

### Differential cytokine responses in patients with COVID-19

**Fig. 3:**
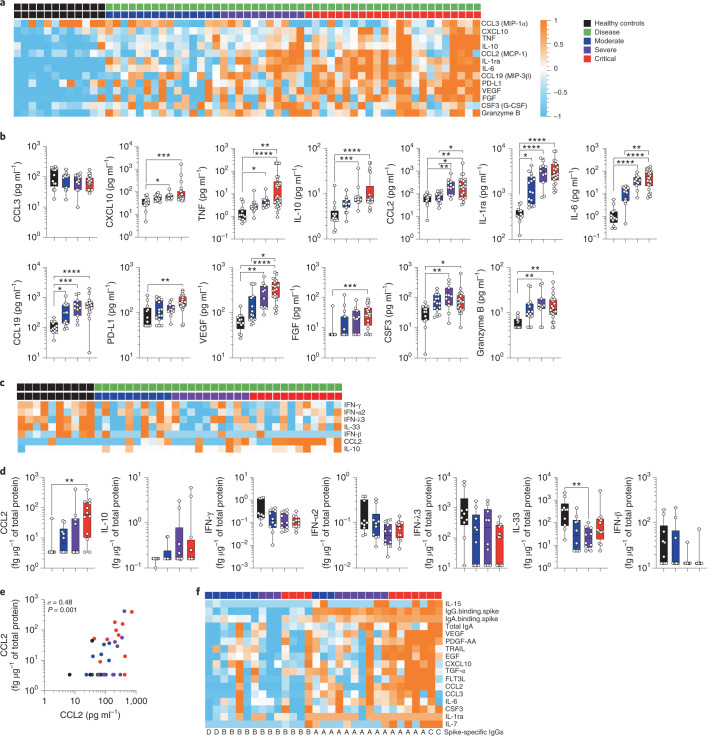
Systemic and mucosal cytokine production in patients with COVID-19. Cytokines were measured in the plasma (**a** and **b**) of healthy controls (*n* = 12 donors) and in patients with mild-to-moderate (*n* = 15), severe (*n* = 11) and critical (*n* = 23) disease or in the nasopharyngeal compartment (**c** and **d**) of healthy controls (*n* = 10 donors) and in patients with mild-to-moderate (*n* = 10), severe (*n* = 10) and critical (*n* = 12) disease using a bead-based multiplexed immunoassay system, Luminex or the digital Simoa ELISA (IFN-α, IFN-β, IFN-γ, IL-6, IL-17A, IL-10 and TNF). **a**,**c**, Heat maps of statistically different cytokines (*P* < 0.05) between healthy controls and patients with COVID-19 (moderate, severe and critical), ordered by hierarchical clustering. Upregulated cytokines are shown in orange and downregulated in blue. **b**,**d**, Individual cytokine concentration plots by patient severity. **e**, Correlation plots between CCL2 concentrations in plasma and nasopharyngeal paired samples; *n* = 42. *σ* represents the Spearman coefficient. **f**, Heat map of statistically different cytokines and antibodies (*P* < 0.05) in patients having nasopharyngeal spike-specific antibodies (type A and type C) as compared with those lacking these antibodies (type B and type D). In **a**, **c** and **f**, *z*-score scale is indicated, with upregulation shown in orange and downregulation shown in blue. *P* values were determined with a two-tailed Mann–Whitney test between healthy and infected individuals. In **b** and **d**, box plots show the median ± minimum to maximum values. *P* values were determined with the Kruskal–Wallis test followed by Dunn’s post hoc test for multiple comparisons. *P < 0.05; **P < 0.01; ***P < 0.001; ****P < 0.0001.

**Extended Data Fig. 3:**
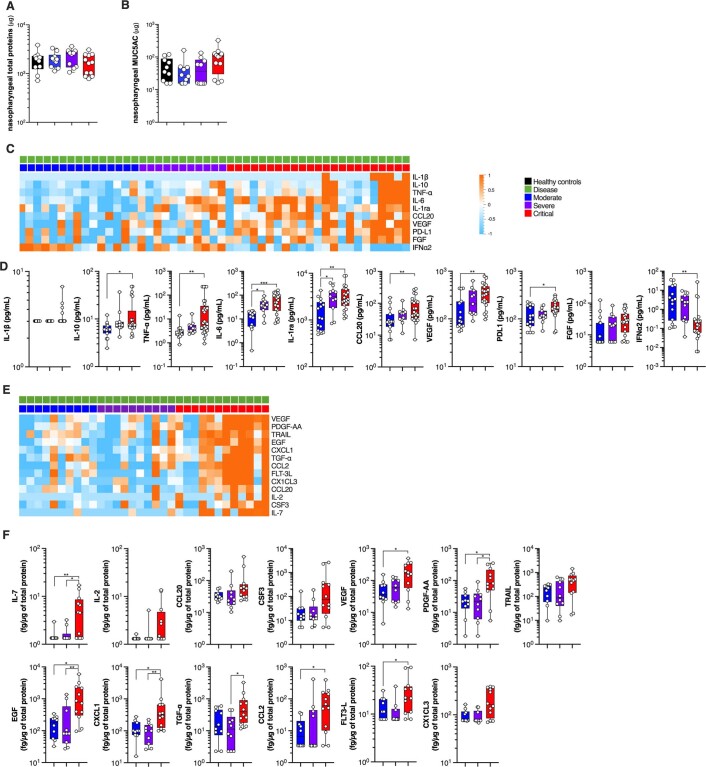
Systemic and mucosal cytokines production in COVID-19 patients. (**a**) total protein (μg) content in nasopharyngeal samples. (**b**) MUC5AC content in nasopharyngeal samples. (**c**) Heatmap representation of statistically different (P < 0.05) plasma cytokines between critical COVID-19 patients and mild to moderate and severe COVID-19 patients. (**d**) Plasma cytokine concentration plots by patient severity. (**e**) Heatmap representation of statistically different (P < 0.05) nasopharyngeal cytokines between critical COVID-19 patients and mild to moderate and severe COVID-19 patients. (**f**) Nasopharyngeal cytokine concentration plots by patient severity. (**a-f**) Cytokines were measured in the plasma of healthy controls (n = 12 donors), mild to moderate (n = 15 patients), severe (n = 11 patients) and critical (n = 23 patients) or in the nasopharyngeal compartment of healthy controls (n = 10 donors), mild to moderate (n = 10 patients), severe (n = 10 patients) and critical (n = 12 patients). In (**c**) and (**e**), P values were determined with the Mann-Whitney test. In (**a**), (**b**), (**d**) and (**f**), box plots with median ± minimum to maximum. P values were determined with the one-sided Kruskal-Wallis test followed by with Dunn’s post-test for multiple group comparisons with Geisser-Greenhouse correction. For all panels: *P < 0.05; **P < 0.01; ***P < 0.001.

To better understand the mechanisms that influence mucosal and systemic spike-specific antibody responses to SARS-CoV-2, we measured the concentrations of 46 cytokines in plasma and nasopharyngeal samples. In plasma, 13 cytokines were significantly different (*P* < 0.05, *q* < 0.2, *n* = 61 samples) between the healthy donors and patients with COVID-19, regardless of disease severity (Fig. 3a,b). These included vascular endothelial growth factor (VEGF), fibroblast growth factor (FGF), IL-1RA, IL-6, TNF, IL-10, CCL2 (MCP-1), CXCL10 (IP-10), CCL3 (MIP-1α), CCL19 (MIP-3β), PD-L1, CSF3 (G-CSF) and granzyme B. In contrast, a strikingly different cytokine profile was observed in the nasopharynx; using the same significance cutoff (*P* < 0.05, *q* < 0.2, *n* = 42 samples), a limited and largely nonoverlapping set of seven cytokines was found to be significantly different between the healthy donors and patients with COVID-19 (Fig. 3c,d). These included IL-33, IFN-α2, IFN-λ3, IFN-β and IFN-γ, which were decreased in the nasopharynx of patients with COVID-19, while IL-10 and CCL2 were increased, as compared to healthy controls (Fig. 3c,d). As protein composition of nasopharyngeal samples may vary between individuals, cytokine measurements were normalized to total protein concentrations^19^. We assessed whether changes in total protein or mucus content might account for the observed differences in nasopharyngeal cytokines. Total protein and MUC5CA levels were not significantly different between controls and patients with COVID-19 in this cohort (Extended Data Fig. 3a,b) and analysis of absolute cytokine levels (without normalization to total protein content) did not affect the results. Of the two plasma and nasopharyngeal cytokines differentially expressed between healthy donors and patients with COVID-19 (IL-10 and CCL2), both were increased during infection in plasma and nasopharyngeal samples (Fig. 3b,d). These results confirm and extend previous reports identifying enhanced inflammatory and diminished interferon responses in the context of SARS-CoV-2 infection^5,20^ but show that nasopharyngeal cytokine responses are regulated in a distinct fashion.

### Cytokine responses stratify COVID-19 disease severity

Previous studies have reported perturbed systemic cytokine production as a hallmark of disease severity in patients with COVID-19 (refs. ^9,21^). Extending our previous work with this cohort^5^, we identified ten circulating cytokines that were significantly different (*P* < 0.05, *q* < 0.2, *n* = 49 samples) between the critical and noncritical (mild-to-moderate and severe) COVID-19 cases. These included IL-6, IL-10, CCL20, VEGF, FGF, PD-L1, TNF, IL-1β and IL-1RA, which increased with disease severity, and IFN-α2, which decreased with severity (Extended Data Fig. 3c,d).

We also studied whether nasopharyngeal cytokine profiles varied with disease severity. Using the same significance cutoff (*P* < 0.05, *q* < 0.2, *n* = 32 samples), we found that 13 nasopharyngeal cytokines were differently regulated between the critical and noncritical COVID-19 cases (Extended Data Fig. 3e,f). Interestingly, only two cytokines (CCL2 and VEGF) overlapped with the plasma dataset (Fig. 3e), whereas other nasal cytokines including FLT3-L, EGF, CXCL1 (GROα), PDGF-AA, IL-7 and TGF-α were significantly increased with worsening disease severity (Extended Data Fig. 3e,f). Taken together, these results suggest that cytokine responses are compartmentalized during SARS-CoV-2 infection and are regulated, similar to spike-specific antibodies, in a tissue-dependent fashion.

As certain cytokines are known to negatively regulate antibody responses (that is, type I interferons^22^), we performed hierarchical clustering of plasma and nasopharyngeal cytokines to identify possible associations that may explain the distinct spike-specific humoral responses (Fig. 2d,e). Analysis of nasopharyngeal cytokines showed higher levels of IL-15, VEGF, PDGF-AA, TRAIL, EGF, CXCL10, TGF-a, FLT3-L, CCL2, CCL3, IL-6, CSF3, IL-1RA and IL-7 in nasotypes A and C (with nasal spike-specific antibodies) compared to nasotypes B and D (without nasal spike-specific antibodies; Fig. 3f), suggesting that inflammation could be involved in local mucosal antibody generation. Further probing of clinical traits using linear regression analysis for quantitative factors and Pearson’s chi-squared tests for qualitative factors, did not identify any clinical signature, other than a positive association (*P* = 0.001) with CRP levels, a trait frequently associated with systemic inflammation^3^. Notably, the interferon response showed no obvious associations with the presence or absence of viral-specific antibodies. These results provide further evidence for distinct host immune responses to SARS-CoV-2 infection at local and systemic levels.

### Viral load drives differential immune responses

**Fig. 4:**
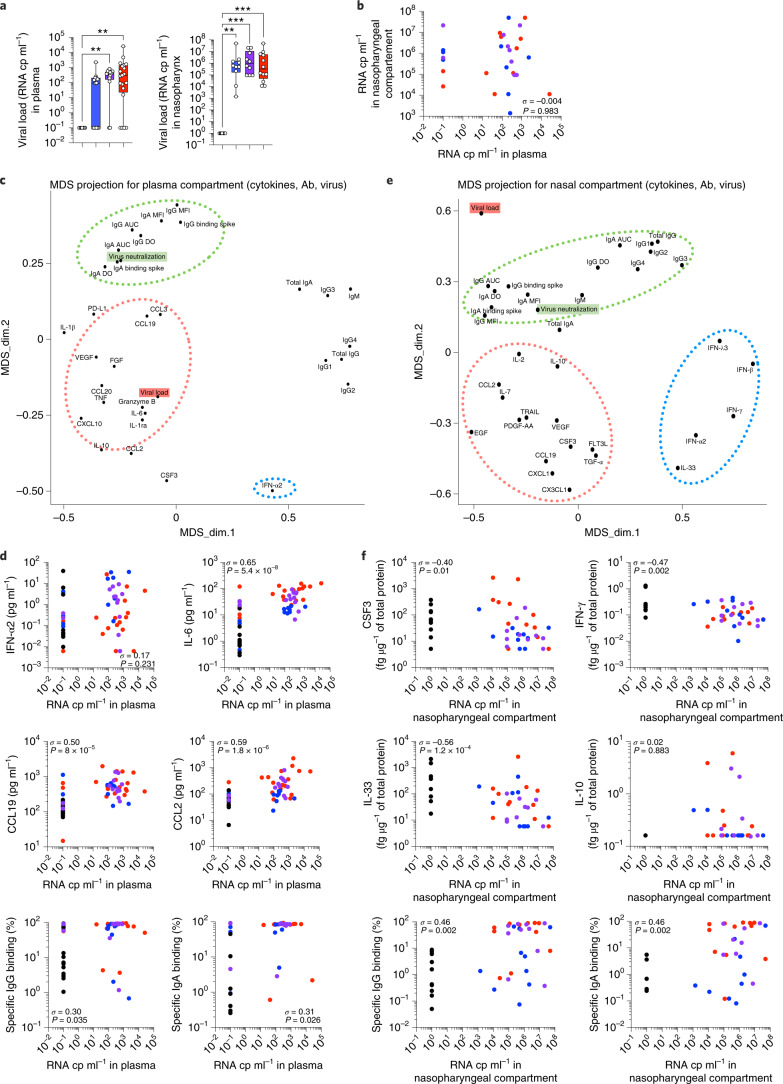
SARS-CoV-2 antiviral immune responses are distinct locally and systemically. **a**, Plasma viral loads evaluated by digital PCR and in nasopharyngeal swabs estimated by RT–PCR and expressed as relative copies (cp) per ml; *n* = 61 (left) and *n* = 42 (right). **b**, Correlation plots between viral load the in the plasma versus nasopharyngeal compartment. **c**,**e**, MDS projection for plasma compartment (cytokines, antibodies and blood viral load; **c**) and nasopharyngeal compartment (cytokines, antibodies and nasal viral load; **e**). The dotted lines represent the most associated analytes. **d**,**f**, Individual correlation plots between viral load and cytokines or antibodies. In **a**, box plots show the median ± minimum to maximum values. *P* values were determined with the Kruskal–Wallis test followed with Dunn’s post hoc test for multiple comparisons. In **b** (*n* = 42), **d** (*n* = 61) and **f** (*n* = 42), *σ* represents the Spearman coefficient. *P < 0.05; **P < 0.01; ***P < 0.001.

**Extended Data Fig. 4:**
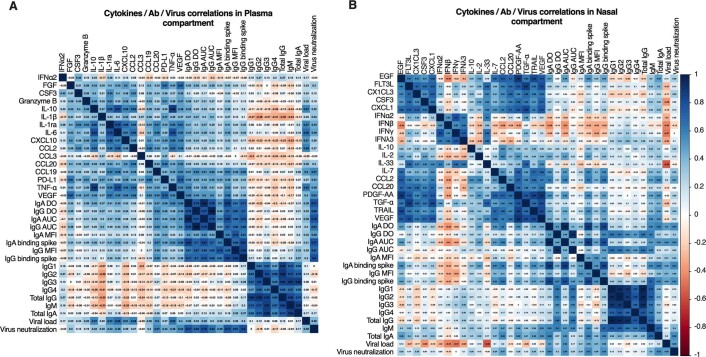
SARS-CoV-2 anti-viral immune responses are distinct locally and systemically. Correlation matrices between the (**a**) systemic compartment (plasma cytokines, plasma antibodies, serum virus neutralization, blood viral load) and (**b**) nasopharyngeal compartment (nasal cytokines, nasal antibodies, nasal virus neutralization, nasal viral load).

We next asked whether the virus may be directly influencing this tissue-specific immunity. We correlated spike-specific antibody and cytokine responses with viral load as measured in nasopharynx by PCR with reverse transcription (RT–PCR), and in plasma with a droplet digital PCR assay as previously described (Methods and ref. ^23^). We found that viral load was increased in both local mucosal and systemic compartments in patients with COVID-19 (Fig. 4a) but were poorly correlated (Fig. 4b). Interestingly, while plasma viral load increased with increasing disease severity, nasopharyngeal viral load was largely independent of the clinical presentation (Fig. 4a), consistent with previous reports^24,25^. To gain insight into how viral load may influence immune responses, we performed multidimensional scaling (MDS), which is a way of visualizing the level of similarity of individual cases of a dataset (in this case viral load, cytokines and antibody response characteristics). From the MDS projection and correlation matrix of our plasma dataset (Fig. 4c and Extended Data Fig. 4a), we could see that viral load was positively associated with the systemic inflammatory response (IL-6, TNF and CCL19) and several regulatory cytokines (IL-10 and IL-1RA) but not with the antiviral interferon response (IFN-α2; Fig. 4c,d). These results are in line with several reports of SARS-CoV-2-dependent induction of hyperinflammation as well as the critical role for interferon responses in controlling initial infection^5,24^. Interestingly, plasma viral load was positioned distinctly from pseudoneutralization activity (forming a cluster with systemic spike-specific IgG and IgA; Fig. 4c). Viral load showed a weak positive correlation with virus-specific antibody responses (Fig. 4d and Extended Data Fig. 4a), suggesting a role for systemic viral load in driving spike-specific humoral immunity.

A similar MDS projection derived from the nasopharyngeal dataset generated a markedly different pattern. In the nasopharynx, SARS-CoV-2 viral loads appeared closely associated with spike-specific IgG and IgA responses (see below). However, in contrast with the plasma, viral loads were not positively associated with any inflammatory or regulatory cytokines and showed strong negative correlations with IL-33, CSF3 and IFN-γ (Fig. 4e,f and Extended Data Fig. 4). These cytokines were decreased in patients with COVID-19 (Fig. 3c,d), suggesting that their loss could be linked to SARS-CoV-2 infection. In addition, disease severity-associated cytokines (EGF, VEGF, FLT3-L, CXCL1, PDGF-AA and TGF-α) clustered away from other variables, indicating distinct regulatory mechanisms (Fig. 4e and Extended Data Fig. 4b).

**Extended Data Fig. 5:**
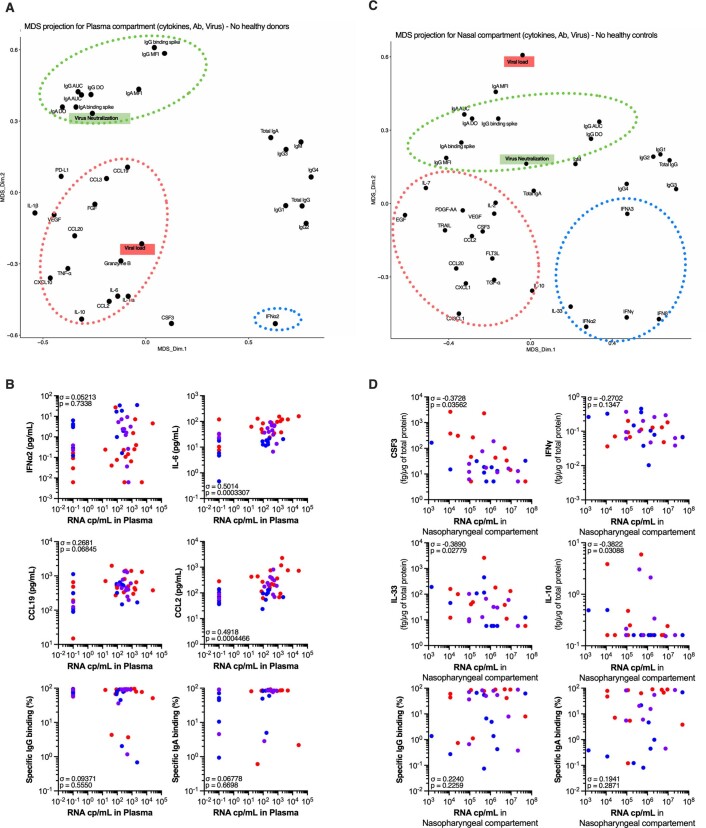
Impact of healthy donors on analysis of integrated anti-viral immune responses. Multidimensional scaling (MDS) projection for (**a**) systemic compartment (plasma cytokines, plasma antibodies, serum virus neutralization, blood viral load) and (**c**) nasopharyngeal compartment (nasal cytokines, nasal antibodies, nasal virus neutralization, nasal viral load). (**b**) and (**d**) Cytokines and viral load were measured in the plasma of healthy controls (n = 12 donors), mild to moderate (n = 15 patients), severe (n = 11 patients) and critical (n = 23 patients) or in the nasopharyngeal compartment of healthy controls (n = 10 donors), mild to moderate (n = 10 patients), severe (n = 10 patients) and critical (n = 12 patients). (**b**) and (**d**) show individual correlation plots between viral load and cytokines or antibodies. In (**b**) and (**d**), σ represents Spearman coefficient and p the p value. For all panels: *P < 0.05; **P < 0.01; ***P < 0.001.

We next assessed whether the observed correlations might be primarily driven by differences between healthy donors and patients, as the former by definition are negative for the virus and lack spike-specific antibodies. We performed the MDS projections of the plasma and nasopharyngeal datasets after exclusion of the healthy donors. The overall MDS projections were quite similar (Extended Data Fig. 5a,c), with plasma viral load driving inflammatory cytokine production (Extended Data Fig. 5b), whereas nasal MDS projections associated viral load inversely with nasopharyngeal cytokines IL-33 and CSF3 (Extended Data Fig. 5d). In contrast, spike-specific antibody correlations were lost in both compartments, indicating that viral load was not the main driver for these responses.

### Nasal microbiome perturbations in SARS-CoV-2

**Fig. 5:**
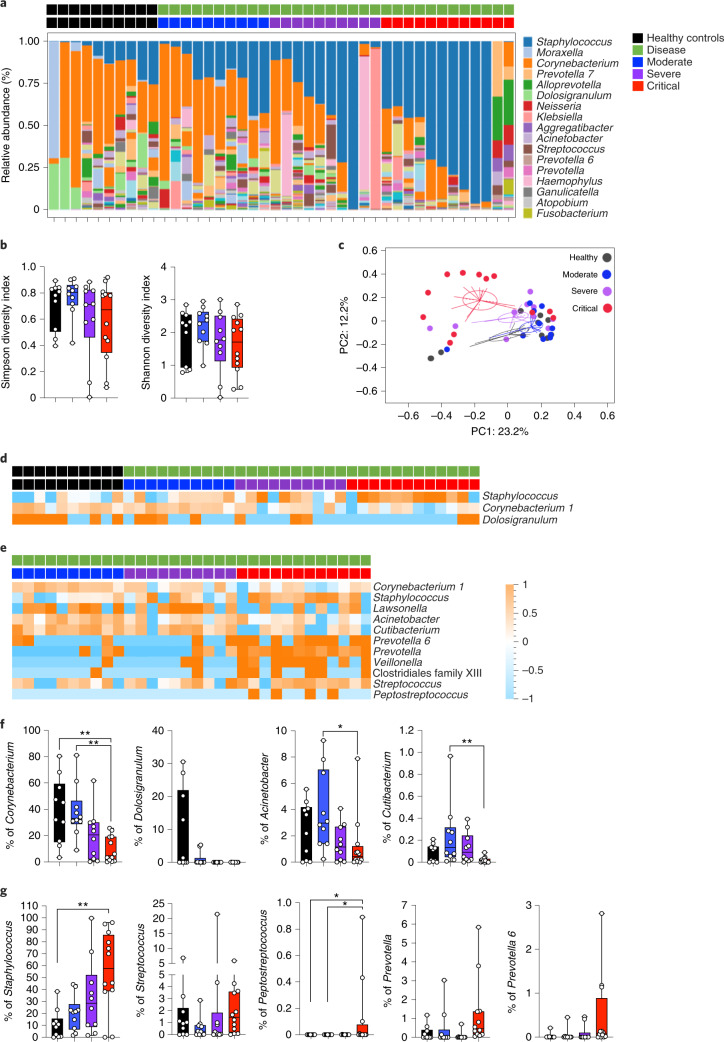
Perturbations of nasopharyngeal 16S rRNA profiles in patients with COVID-19. **a**–**g**, Nasopharyngeal bacterial communities were measured in healthy controls (*n* = 10) and in patients with mild-to-moderate (*n* = 10), severe (*n* = 10) and critical (*n* = 12) COVID-19. **a**, The percentage of relative abundance at the genus level. **b**, Shannon and Simpson diversity indices by patient severity. Data are presented as box plots with median ± minimum to maximum. **c**, Principal-component analysis of 16S bacterial profiles. **d**, Heat map of statistically different (*P* < 0.05) genus abundance between healthy controls and patients with COVID-19 (moderate, severe and critical). **e**, Heat map of statistically different (*P* < 0.05) genus abundance between patients with COVID-19 depending on disease severity. *P* values were determined with a two-tailed Mann–Whitney test. **f**,**g**, Plots showing the percentage of individual genus abundance by disease severity. In **b**, **f** and **g**, box-and-whisker plots show the minimum and maximum values, interquartile range and the median. *Corynebacterium* (critical versus healthy, *P* = 6.9 × 10^−^^3^; critical versus moderate, *P* = 2.6 × 10^−^^3^), *Acinetobacter* (critical versus moderate, *P* = 3.2 × 10^−^^2^), *Cutibacterium* (critical versus moderate, *P* = 6.6 × 10^−^^3^), *Staphylococcus* (critical versus healthy, *P* = 9.0 × 10^−^^3^), *Peptostreptococcus* (critical versus healthy, *P* = 3.3 × 10^−^^2^; critical versus moderate, *P* = 3.3 × 10^−^^2^). *P* values were determined with the one-sided Kruskal–Wallis test followed by Dunn’s post hoc test for multiple comparisons with Geisser–Greenhouse correction; *P < 0.05; **P < 0.01; ***P < 0.001. In **e**, *z*-score scale is indicated, with upregulation shown in orange and downregulation shown in blue.

The upper respiratory tract harbors diverse microbial commensal communities that are implicated in protection against disease-causing pathogens^26^. We hypothesized that perturbations in nasopharyngeal microbial profiles might contribute to the diverse outcomes of immune responses and clinical presentation during SARS-CoV-2 infection. We performed unbiased bacterial 16S rRNA sequencing to better characterize the commensal communities and potential pathobiont carriage in the nasopharynx of controls and patients with COVID-19 (*n* = 42). V3–V4 region amplicons were sequenced and analyzed using SHAMAN^27^ allowing for identification of 464 operational taxonomic units (OTUs). Genus-level analysis demonstrated significant (*P* < 0.05) perturbations comparing healthy controls to patients with COVID-19 (Fig. 5a). In addition, analysis of α-diversity (Simpson and Shannon diversity indices; combined measures of evenness and number of bacteria) showed a decrease in 16S rRNA sequences in patients with severe and critical COVID-19 (Fig. 5b). Richness of microbiota communities (β-diversity) clearly decreased with disease severity and an analysis based on Bray–Curtis distance matrix and subjected to principal-coordinate analysis suggested that 16S rRNA profiles in patients with critical disease were different from other patients (Fig. 5c). To substantiate these observations, we performed a parallel analysis using DADA2 (ref. ^28^), allowing for the identification of 351 amplicon sequence variants (ASVs). Comparisons of SHAMAN and DADA2 pipelines revealed similar annotation profiles (SHAMAN: family 96.7%, genus 80.8%, species 33.9%; versus DADA2: family 98.0%, genus 88.9%, species 21.7%) and a higher mapping rate for SHAMAN (average 79% ± 16% for OTUs versus 72% ± 19% for ASVs, *P* = 2.19 × 10^−11^ using a paired *t*-test).

**Extended Data Fig. 6:**
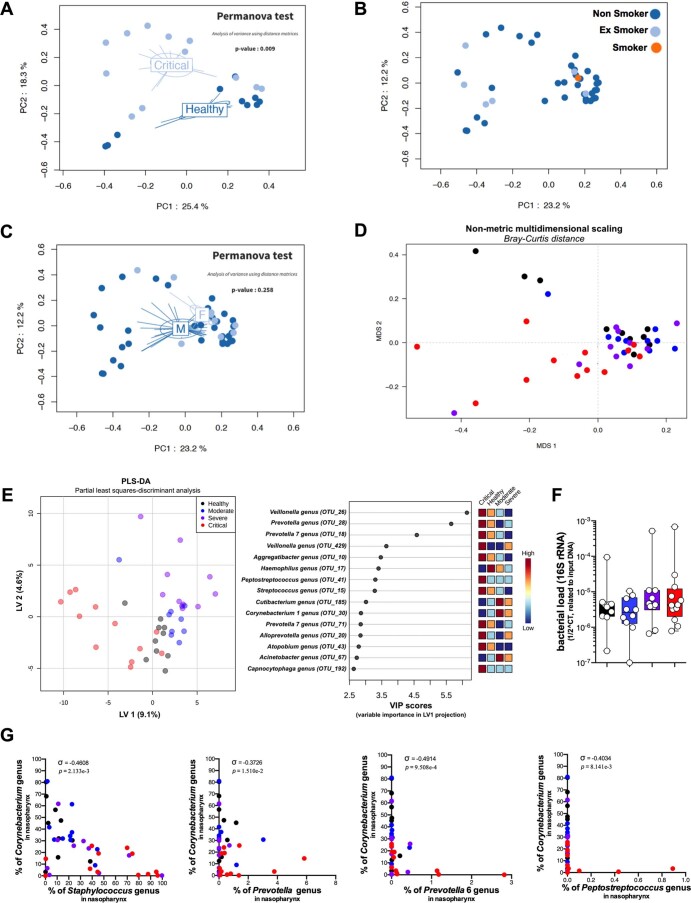
Perturbations of nasopharyngeal 16 S rRNA profiles in COVID-19 patients. PCA and PERMANOVA test analysis of 16 S bacterial profiles of healthy versus critical COVID-19 patients. PCA analysis of 16 S bacterial profiles color coded by smoking status (**b**) and sex (**c**). (D) Non-metric multidimensional scaling (MDS) of 16 S bacterial profiles. (**e**) Partial least squares-discriminant analysis of 16 S bacterial profiles (**f**) Bacterial load (16 S rRNA) plotted by patient severity. Box plots with median ± minimum to maximum. (**g**) Individual correlation plots between Genus abundance (%) for ‘cornerstone’ and ‘pathobionts’. (**f**) and (**g**) Bacterial communities were measured in the nasopharyngeal compartment of healthy controls (n = 10 donors), mild to moderate (n = 10 patients), severe (n = 10 patients) and critical (n = 12 patients). In (**g**), σ represents Spearman coefficient and p the p value. In (F), P values were determined with the one-sided Kruskal-Wallis test followed by with Dunn’s post-test for multiple group comparisons with Geisser-Greenhouse correction. For all panels: *P < 0.05; **P < 0.01; ***P < 0.001.

As a permutational multivariate analysis of variance test showed that nasopharynx microbiota of patients with critical disease is significantly different from that of healthy controls (Extended Data Fig. 6a), we searched for clinical signatures that might help explain these differences. Smoking and sex did not affect this clustering (Extended Data Fig. 6b,c). Moreover, nasopharyngeal samples were obtained before any antibiotic treatment in the clinic. These results suggest that SARS-CoV-2 infection might induce perturbations in nasopharyngeal microbial communities.

**Extended Data Fig. 7:**
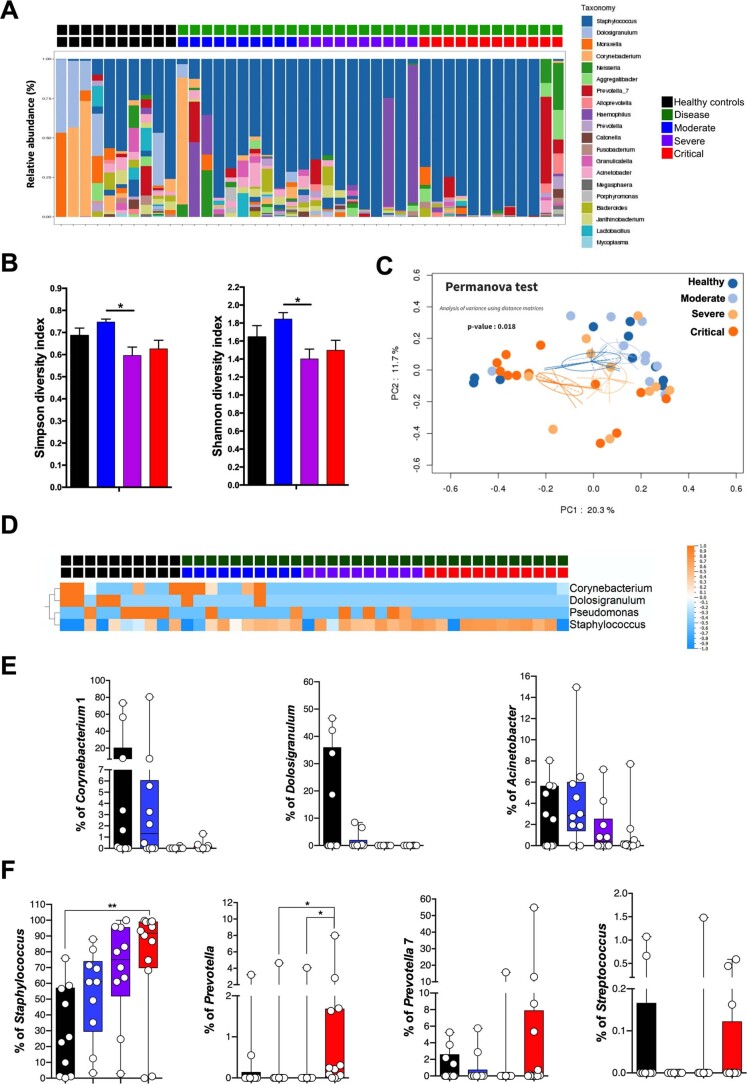
16 S rRNA microbiome analysis in COVID-19 patients using DADA2 pipeline. Nasopharyngeal bacterial communities were measured in healthy controls (n = 10), mild to moderate (n = 10), severe (n = 10) and critical (n = 12) COVID-19 patients. (**a**) Relative abundance (%) at the Genus level. (**b**) Shannon and Simpson diversity indices by patient severity. Data are presented as mean values + /- SEM. Simpson diversity index (Severe vs Moderate, p = 2.1 × 10-2), Shannon diversity index (Severe vs Moderate, p = 4.1 × 10-2), (**c**) PCA analysis of 16 S bacterial profiles (**d**) Heatmap representation of statistically different (P < 0.05) Genus abundance between healthy controls and COVID-19 patients (moderate, severe, critical). P values were determined with the Mann-Whitney test. (**e**) and (**f**) Individual Genus abundance (%) plots by disease severity. For all plots, bacterial communities were measured in the nasopharyngeal compartment of healthy controls (n = 10 donors), mild to moderate (n = 10 patients), severe (n = 10 patients) and critical (n = 12 patients). In (**b**), (**e**) and (**f**), box-and-whisker plots showing the minimum, maximum, interquartile range and the median. Staphylococcus (Critical vs Healthy, p = 6.5 × 10-3), Prevotella (Critical vs Moderate, p = 2.7 × 10-2; Critical vs Severe, p = 2.5 × 10-2). P values were determined with the one-sided Kruskal-Wallis test followed by with Dunn’s post-test for multiple group comparisons with Geisser-Greenhouse correction; For all panels: *P < 0.05; **P < 0.01; ***P < 0.001.

We applied a nonmetric multidimensional scaling using Bray–Curtis distances and partial least-squares discriminant analysis, which identified patients with critical disease as different from other patients with COVID-19 (Extended Data Fig. 6d,e). While nasopharyngeal bacterial load did not change (Extended Data Fig. 6f), specific genera showed significant differences (*P* < 0.05) between patients and healthy controls (Fig. 5d,e), including *Corynebacterium* and *Dolosigranulum*, that are thought to provide protection against pathogen and pathobiont invasion (‘beneficial’ commensals)^26^. These were markedly reduced in patients with COVID-19 in a severity-dependent fashion (Fig. 5d,f). In contrast, the *Staphylococcus* genus and several strict anaerobes (including *Peptostreptococcus* and *Prevotella* genera) were increased in patients with critical COVID-19 (Fig. 5e,g). A parallel analysis using DADA2 pipelines confirmed these microbiome changes in patients with severe and critical COVID-19 (Extended Data Fig. 7). These results demonstrate that SARS-CoV-2 infection is associated with perturbations in nasopharyngeal bacterial communities and with accompanying dysbiosis in patients with critical COVID-19, although we cannot rule out the possibility that some characteristics of this ‘dysbiosis’ were already present in individuals before SARS-CoV-2 infection.

**Fig. 6:**
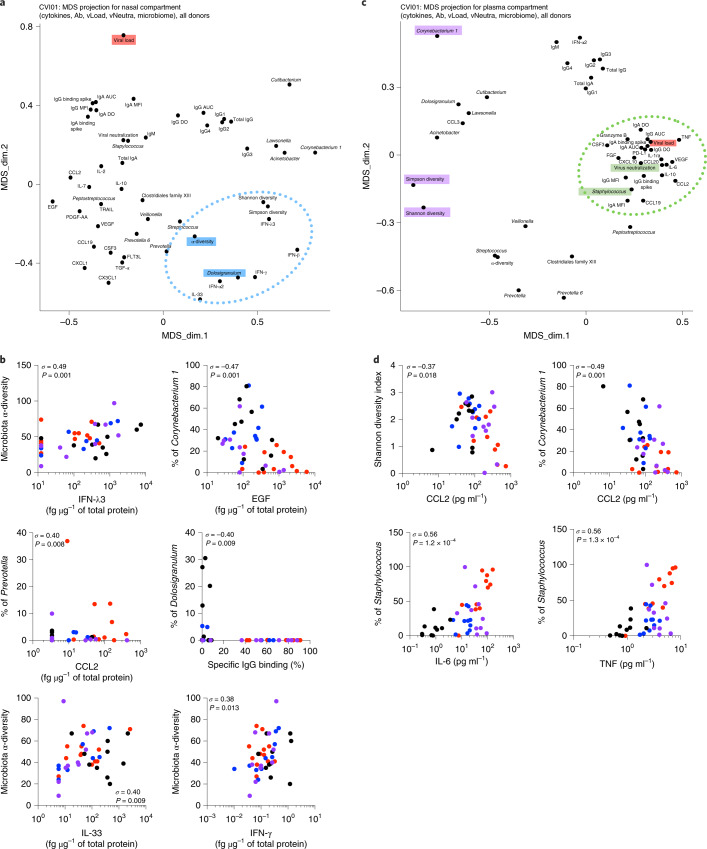
Nasal microbiome influences local mucosal and systemic immune responses in patients with COVID-19. **a**,**c**, DS projection for the nasopharyngeal compartment (cytokines, antibodies, neutralization, nasal viral load and nasal microbiome; **a**) and the plasma compartment (cytokines, antibodies, pseudoneutralization, blood viral load and nasal microbiome; **c**). The dotted lines represent the most associated analytes. **b**,**d**, Plots show individual correlations between the percentage of genus abundance and cytokines, antibodies or viral load. In **b** (*n* = 42) and **d** (*n* = 61), *σ* represents the Spearman coefficient. *P < 0.05; **P < 0.01; ***P < 0.001.

**Extended Data Fig. 8:**
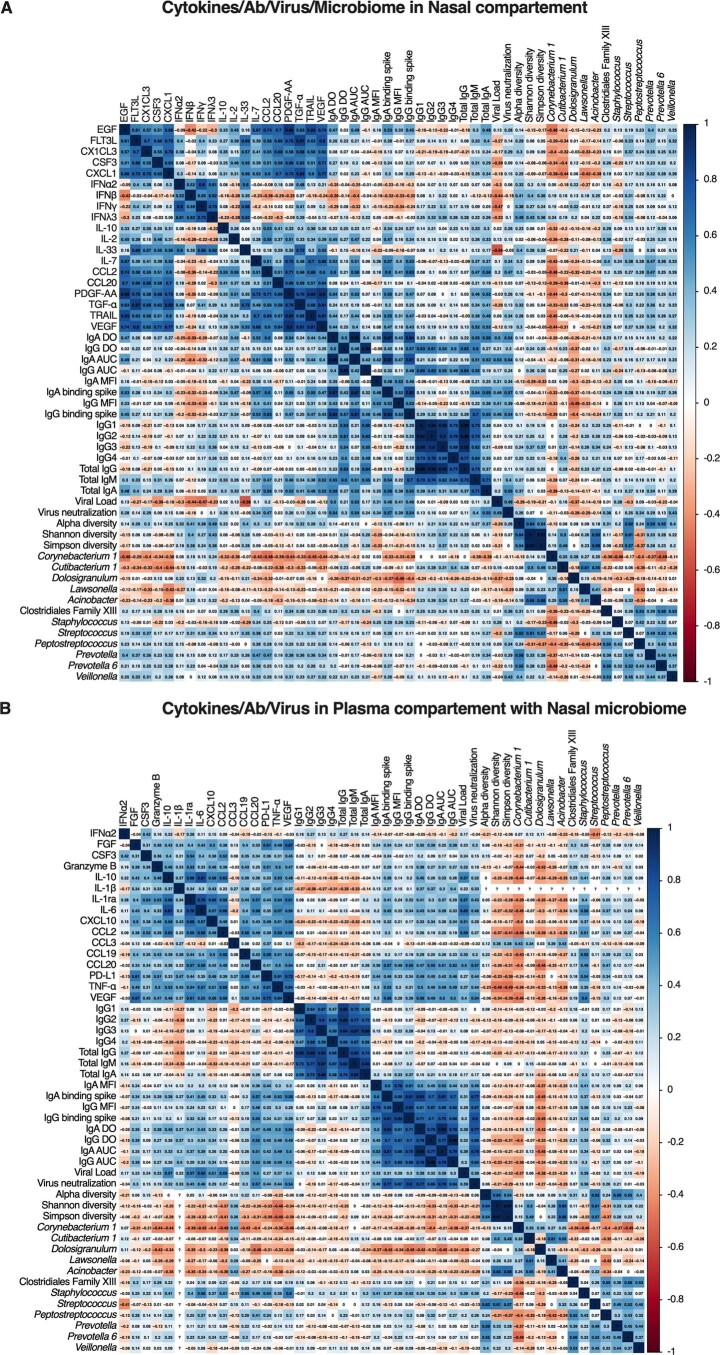
Microbiome regulates mucosal cytokines and antibody responses. Correlation matrices between the (**a**) nasopharyngeal compartment (nasal cytokines, nasal antibodies, nasal virus neutralization, nasal viral load and nasal microbiome) and (**b**) systemic compartment (plasma cytokines, plasma antibodies, serum virus neutralization, blood viral load and nasal microbiome).

**Extended Data Fig. 9:**
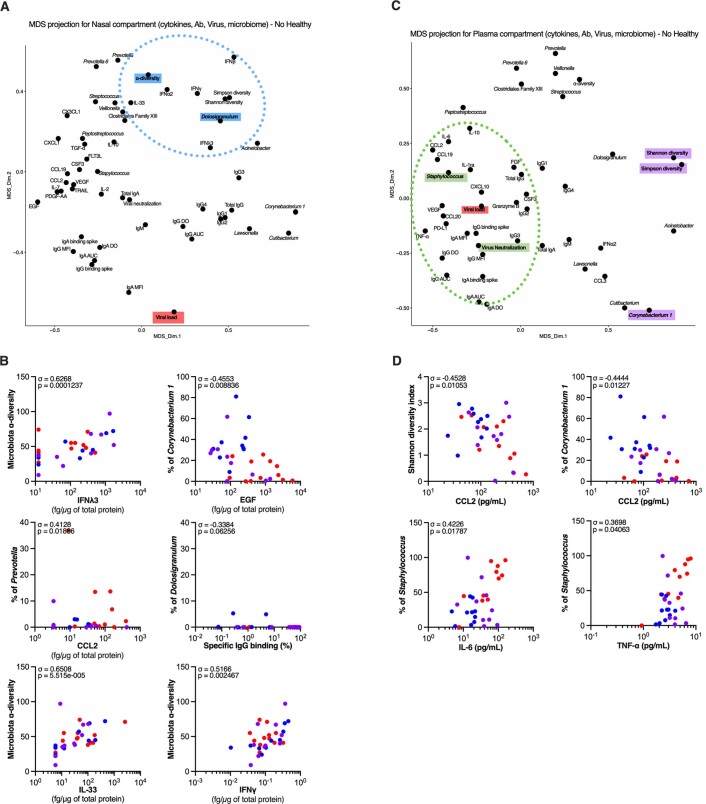
Impact of healthy donors on microbiome analysis of integrated SARS-CoV-2 immune responses. Multidimensional scaling (MDS) projection for (**a**) nasopharyngeal compartment (nasal cytokines, nasal antibodies, nasal virus neutralization, nasal viral load and nasal microbiome) and (**c**) systemic compartment (plasma cytokines, plasma antibodies, serum virus neutralization, blood viral load and nasal microbiome). The dotted lines represent the most associated measures. (**b**) and (**d**) show individual correlation plots between Genus abundance (%) and cytokines and specific antibodies. (**b**) and (**d**) Cytokines and viral load were measured in the plasma of healthy controls (n = 12 donors), mild to moderate (n = 15 patients), severe (n = 11 patients) and critical (n = 23 patients) or in the nasopharyngeal compartment of healthy controls (n = 10 donors), mild to moderate (n = 10 patients), severe (n = 10 patients) and critical (n = 12 patients). In (**b**) and (**d**), σ represents Spearman coefficient and p the p value. For all panels: *P < 0.05; **P < 0.01; ***P < 0.001.

**Extended Data Fig. 10:**
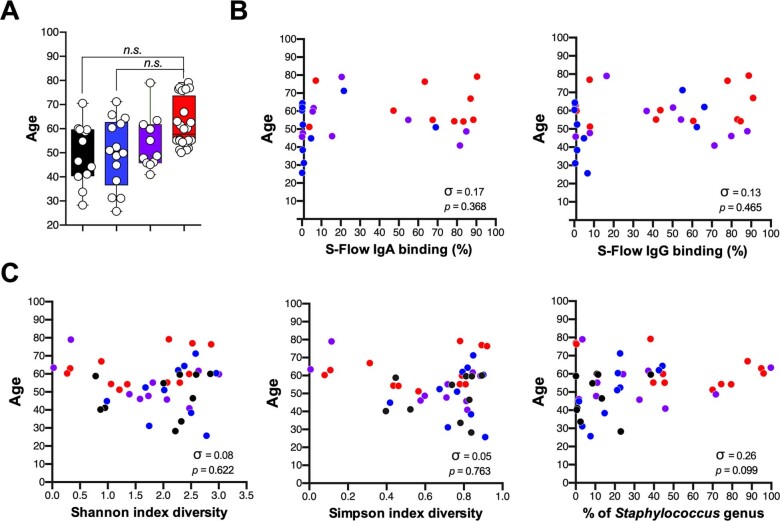
Influence of age on integrated SARS-CoV-2 immune responses. (**a**) Age distribution of the cohort based on disease severity. Box plots with median ± minimum to maximum. (**b**) Correlation plots between age and nasal S-Flow anti-Spike IgG or IgA binding (%). (**c**) Correlation plots between age and nasopharyngeal microbiota Shannon and Simpson diversity and Staphylococcus genus abundance (%). For all plots, analytes were measured in the plasma of healthy controls (n = 12 donors), mild to moderate (n = 15 patients), severe (n = 11 patients) and critical (n = 23 patients) or in the nasopharyngeal compartment of healthy controls (n = 10 donors), mild to moderate (n = 10 patients), severe (n = 10 patients) and critical (n = 12 patients).In (**b, c**), σ represents Spearman coefficient and p the p value. For all panels: *P < 0.05; **P < 0.01; ***P < 0.001.

Finally, we integrated the 16S rRNA bacterial nasopharyngeal microbiome profiles with the immune response (spike-specific antibodies and cytokines) and performed MDS projections in an attempt to undercover associations that might explain mechanistic relationships at this mucosal site. Interestingly, cytokines that decreased (IL-33, IFN-λ3 and IFN-γ) or increased (EGF) with SARS-CoV-2 infection were linked to overall microbial α-diversity and to presence of *Corynebacterium* (Fig. 6a,b and Extended Data Fig. 8a) suggesting genus-specific and community-driven regulation of mucosal cytokine production. Nasopharyngeal viral load more closely associated with *Staphylococcus* genus abundance, whereas other potential nasopharyngeal pathobionts (including *Prevotella*, *Streptococcus*, *Peptostreptococcus* and *Clostridial* genera) were linked to disease severity-associated nasopharyngeal cytokines (CCL2 and VEGF; Fig. 6a,b and Extended Data Fig. 8a). MDS projections after integrating nasal microbiome profiles into the plasma datasets revealed intriguing associations of microbiome features with systemic viral load, spike-specific responses, neutralization capacity and inflammatory cytokines, including positive correlations of *Staphylococcus* with inflammatory cytokines (IL-6 and TNF) and negative correlations of microbial diversity and *Corynebacterium* with CCL2 (Fig. 6c,d and Extended Data Fig. 8b). MDS projections obtained after exclusion of healthy donors maintained these associations and correlations supporting their potential role in COVID-19 disease severity (Extended Data Fig. 9a–d). Finally, age differences could be ruled out as an important driver of severity-associated immune and microbiome phenotypes (Extended Data Fig. 10). Taken together, these results reveal an unexpected relationship between nasopharyngeal microbial communities and local as well as systemic, cytokine and antibody responses during SARS-CoV-2 infection.

## Discussion

Despite widespread studies, we still lack a full understanding of how local and systemic immune responses are dysregulated following SARS-CoV-2 infection and the individual roles that they play in determining severe clinical outcomes in a minority of patients with COVID-19. To address this question, we compared systemic and local immune responses during active SARS-CoV-2 infection in a well-characterized COVID-19 cohort. We measured host antibody and cytokine responses, determined viral load and characterized the nasopharyngeal 16S rRNA profiles using an integrative approach. Comparative analysis of the systemic and local tissue responses suggests a model for protective immunity following SARS-CoV-2 infection and identifies potential regulatory nodes where perturbations may lead to more severe COVID-19 clinical manifestations. First, a healthy nasopharyngeal microbiome (harboring, for example, ‘beneficial’ components that confer colonization resistance) appears linked to production of nasal cytokines including IL-33, IFN-γ, IFN-α/β and IFN-λ3. SARS-CoV-2 infection, either directly or indirectly, appears to disrupt local microbial homeostasis, resulting in reduced levels of these cytokines that may be important for viral control. Second, while viral load impacts specific humoral immune responses, the local cytokine milieu is also important as evidenced by weaker nasopharyngeal antibody responses in individuals that have lower levels of mucosal inflammatory cytokines. Third, a relative increase in certain bacterial genera associate with enhanced mucosal and systemic inflammation, mediated through distinct cytokine profiles, and correlate with worsening clinical outcome. Together, these findings raise several key questions regarding host mechanisms that can enhance resistance to SARS-CoV-2 infection and associated clinical manifestations.

Resistance to infection by bacterial, fungal and/or viral pathogens is in part mediated through commensal microbial communities that inhabit mucosal surfaces. Several ‘cornerstone’ members contribute to this effect, including *Corynebacterium*, *Dolosigranulum*, *Cutibacterium*, *Lactobacillus* and other genera that generate a ‘frontline’ defense against de novo infection and suppress progression of ‘pathobionts’ that are present as carriage in normal healthy individuals^26,29^. The mechanisms for this microbial resistance vary and include stimulation of mucous layers and elaboration of antimicrobial peptides^26,29^. The participation of commensal communities in antiviral defense is poorly defined, but our results suggest that they may be involved in maintenance of basal production of interferon type I, II and III. Previous studies suggest that microbiota control the constitutive production of type I and type III interferons^30–32^ and modulate the resistance to virus infections in mice^33,34^. A recent report found decreased microbial diversity in patients with COVID-19 (ref. ^35^), which we can link to reduced cytokine levels in the nasopharyngeal compartment. Individual variation in microbiome-dependent interferon levels may in part provide an explanation for the differential outcome (resistance versus productive infection and potential spread) after SARS-CoV-2 encounter.

Analysis of the nasopharyngeal antibody response also revealed highly heterogenous responses. While the vast majority of patients generated systemic viral-specific antibodies, a surprisingly high proportion of patients had neither detectable viral-specific IgG nor IgA in their nasopharyngeal compartments despite the use of highly sensitive assays. The presence of nasopharyngeal antibodies appeared to be primarily regulated by local inflammation and cytokine production with little evidence for a role of viral load. As our study relied on a single sample (taken on days 8–12 after symptom onset), we cannot exclude that later ‘naso-conversion’ will occur in patients lacking mucosal spike-specific antibodies. Additional studies involving replication cohorts and longitudinal sampling may be required to determine mechanistic interactions. Recently IgA, and in particular dimeric IgA, were shown to have the most potent neutralizing activity against SARS-CoV-2 infection^36^ especially in the early phase of infection^14^. Understanding mechanisms that allow for efficient upregulation of local IgA production (as in type C individuals) and local viral control^37^ may provide new avenues for protection against SARS-CoV-2. In light of these and our own findings, the nasopharyngeal immune response should be considered as a potential biomarker for correlates of protection during SARS-CoV-2 vaccination campaigns.

Multiple studies have recently described a parallel impaired type I interferon activity and exacerbated inflammatory cytokine response in severe COVID-19 disease^5^. While systemic hyperinflammation is likely to be detrimental for an uneventful clinical recovery, such responses may be required during initial infection, as reflected by the poor outcome of clinical studies targeting these cytokine pathways (anti-IL-6 and IL-1 therapies) during the early phase of disease. Furthermore, while the importance of type I interferons has been demonstrated through multiple lines of evidence, including both genetic variants and the presence of neutralizing antibodies in patients with severe disease^20,38^, some uncertainty remains in the literature possibly due to differences in the site of analysis, methods used or the time point studied^21,39,40^, as well as their lack of efficacy in randomized placebo control trials^41^. As such, comparative in-depth analyses of local and systemic inflammatory cytokines are warranted to uncover potential mechanisms that might regulate disease severity in patients with COVID-19. The main overlap between mucosal and systemic compartments was an increase in CCL2, a critical cytokine for recruitment of monocytes to infected and inflamed tissues. These findings support previous reports indicating a general critical role for this cytokine in COVID-19 disease^21,42^, which was elevated in bronchoalveolar lavage fluid from the lungs of patients with COVID-19 during mechanical ventilation^43^. Furthermore, a recent genome-wide association study analysis identified a polymorphism in the CCL2 receptor (CCR2) as associated with critical COVID-19 illness^44^.

However, the main striking result from our study was a notable elevation in patients with critical illness of a cluster of plasma cytokines and growth factors that did not have an obvious role in antiviral immunity. Insight into their implication in severe COVID-19 illness came from the integration of plasma datasets with the nasal microbiome, which revealed positive associations with opportunistic bacterial genera such as *Prevotella* and *Streptococcus*, and negative associations with key mucosal cornerstone genera such as *Corynebacterium* and *Dolosigranulum*. This hypothesis was supported by additional associations between presence of nasopharyngeal *Staphylococcus* genus and plasma inflammatory cytokines such as IL-6. A recent report found an association of *Prevotella* with COVID-19 disease severity^45^ that we can link to systemic levels of inflammatory cytokines. Whether such nasal dysbiosis drives systemic inflammation will require further study, but this hypothesis is further supported by a recent study documenting a similar mechanism in the infected intestine^46^. Alternatively, nasopharyngeal ‘dysbiosis’ may precede SARS-CoV-2 infection rendering these individuals more susceptible to COVID-19 morbidities. Previous studies have documented ‘pathobiont’ carriage (including *Staphylococcus aureus*, *Streptococcus pneumoniae* and *Haemophilus influenzae*) in up to 40% of healthy individuals^26^. Our results suggest that these individuals may be at higher risk of developing severe COVID-19 disease, as SARS-CoV-2 infection would result in a breakdown of local epithelial barrier function leading to escape of these potential pathobionts with resultant systemic manifestations. In summary, our study identifies new host–viral–microbiome interactions during infection with SARS-CoV-2, which may help to uncover new strategies for identifying at-risk individuals.

## Methods

### Study design

This non-interventional study was conducted between 19 March 2020 and 3 April 2020 in Cochin Hospital (Paris, France), in the setting of the local RADIPEM biological samples collection, derived from samples collected in routine care as previously described^5^. Biological collection and informed consent were approved by the Direction de la Recherche Clinique et Innovation and the French Ministry of Research (no. 2019-3677). Inclusion criteria for COVID-19 inpatients were: aged between 18 and 80 years, diagnosis of COVID-19 according to WHO (World Health Organization) interim guidance, and positive SARS-CoV-2 RT–PCR testing on a respiratory sample (nasopharyngeal swab or invasive respiratory sample). Inpatients with preexisting unstable chronic disorders (such as uncontrolled diabetes mellitus, severe obesity defined as body mass index greater than 30, unstable chronic respiratory disease or chronic heart disease) and with bacterial co-infection were excluded. Because median duration from onset of symptoms to respiratory failure was previously shown to be 9.5 (interquartile range, 7.0–12.5) days^47^, we analyzed immune responses between 8 to 12 d after onset of first symptoms for all patients and before the initiation of any antiviral or anti-inflammatory treatment. Healthy controls were asymptomatic adults, matched with individuals with COVID-19 on age (±5 years), who had a negative SARS-CoV-2 RT–PCR test at the time of inclusion. The study conforms to the principles outlined in the Declaration of Helsinki, and received approval by the appropriate Institutional Review Board (Cochin-Port Royal Hospital, Paris; no AAA-2020–08018).

Epidemiological, demographic, clinical, laboratory, treatment and outcome data were extracted from electronic medical records using a standardized data collection form. Chest radiographs or computed tomography (CT) scan were also done for all inpatients. Laboratory confirmation of SARS-CoV-2 was performed at the Cochin Hospital. RT–PCR assays were performed in accordance with the protocol established by the WHO (COVID-19 technical guidance: laboratory testing for 2019-nCoV in humans; https://www.who.int/publications/i/item/10665-331501).

The severity of COVID-19 was classified at the time of admission based on the adaptation of the Sixth Revised Trial Version of the Novel Coronavirus Pneumonia Diagnosis and Treatment Guidance. Mild cases were defined as mild clinical symptoms (fever, myalgia, fatigue and diarrhea) and no sign of pneumonia on thoracic CT scan. Moderate cases were defined as clinical symptoms associated with dyspnea and radiological findings of pneumonia on thoracic CT scan, and requiring a maximum of 3 l min^−1^ of oxygen, stable for at least the following 24 h. Severe cases were defined as respiratory distress requiring more than 3 l min^−1^ of oxygen and no other organ failure, stable for at least the following 24 h. Critical cases were defined as respiratory failure requiring mechanical ventilation, shock and/or other organ failure that require an intensive care unit (ICU).

### Patient characteristics

Forty-nine patients with COVID-19 and twelve healthy controls were included. The demographic and clinical characteristics of the patients have been previously described^5^. The median age of the patients was 55 years (interquartile range, 50 to 63 years) and 78% were male, while the median age of healthy controls was 51 years (interquartile range, 38 to 60 years) and 72% were male. Patients were sampled for plasma and nasopharyngeal swabs after a median duration of 10 d (interquartile range, 9 to 11 d) after disease onset. Fever was present in 98% of the patients, and the other most common symptoms were dyspnea (98%), fatigue (96%), cough (92%), myalgia (62%) and diarrhea (34%). Among the whole population, 44% had at least one controlled coexisting illness, mainly hypertension and type 2 diabetes.

On admission, the degree of severity of COVID-19 was categorized as moderate in 15 patients (median oxygen requirement 2 l min^−1^), severe in 11 patients (median oxygen requirement 5 l min^−1^) and critical in 23 patients. Of the CT scans available at the time of admission, all were abnormal, showing ground-glass opacities (100%) with bilateral patchy distribution (96%). Most of the patients had elevated CRP, ferritin and LDH levels. Patients with severe and critical disease had more prominent laboratory abnormalities than those with mild-to-moderate disease, and extension on chest CT scan was correlated with disease severity. No patients with moderate disease required admission to an ICU or the use of mechanical ventilation, while 6 of 11 patients with severe disease were eventually admitted to the ICU.

### Nasopharynx swab processing

Nasopharynx swabs were thawed in a P3 laboratory and vortexed for 1 min at 2,500 r.p.m. to ensure complete sample recovery. Samples (1 ml medium) were transferred in a 96-well deep-well plate and centrifuged at 16,000*g* for 10 min at 4 °C to pellet the cells and accompanying microbes for 16S rRNA-sequencing analysis. Supernatants were recovered and either heat inactivated for antibody analysis, or treated for cytokine analysis as described below. Total protein determinations were performed using the Bio-Rad Protein Assay^48^ with serum albumin as standard.

### Antibody assays

SARS-CoV-2-specific antibodies were quantified using assays previously described^16^. Briefly, a standard ELISA assay (data collected with the Multiskan Spectrum; Thermo Fisher Scientific), using as target antigens the extracellular domain of the spike protein in the form of a trimer (ELISA tri-S), and the S-Flow assay, which is based on the recognition of SARS-CoV-2 spike protein expressed on the surface of 293T cells (293T-S), were used to quantify SARS-CoV-2-specific IgG and IgA subtypes in plasma and nasopharyngeal swab supernatants. Briefly, specific IgG and IgA were detected in S-Flow assay by anti-IgG Alexa Fluor 647 (A-21445, Thermo Fisher Scientific, polyclonal; dilution 1:600) or anti-IgA Alexa Fluor 647 (109-605-011, Jackson ImmunoResearch, polyclonal; dilution 1:800). S-Flow assay is a flow cytometry-based assay. The data were acquired with an AttuneTM NxT v3.2.1243.0 and analyzed with FlowJo v10. Assay characteristics including sensitivity and specificity were previously described^16^. Total IgA, IgM, IgG1, IgG2, IgG3 and IgG4 were determined using the Bio-Plex Pro Human Isotyping Assay Panel (Bio-Rad) according to the manufacturer’s instructions. Data were acquired on a Bio-Plex 200 system (Bio-Rad) and analyzed using Bio-Plex Manager v5 (Bio-Rad).

### Cytokine and mucin assays

Before protein analysis, plasma and nasal samples were treated in a P3 laboratory for viral decontamination using a protocol previously described for SARS-CoV^49^, which we validated for SARS-CoV-2. Briefly, samples were treated with 1% TRITON X100 (vol/vol) and 0.3% tri-*N*-butyl phosphate (vol/vol) for 2 h at room temperature. Tri-*N*-butyl phosphate was removed before cytokine analysis by passing the treated samples though C18 columns. IFN-α2, IFN-γ and IL-17A (triplex) and IFN-β and IFN-λ3 (both single plex) protein plasma and nasopharyngeal concentrations were quantified by Simoa assays developed with Quanterix Homebrew kits as previously described^50^. Data were collected with a Simoa HD-1 analyzer (Quanterix). IL-6, TNF and IL-10 were measured with a commercial triplex assay (Quanterix). For the IFN-α2 assay, the BMS216C (eBioscience) antibody clone was used as a capture antibody after coating on paramagnetic beads (0.3 mg ml^−1^), and the BMS216BK already biotinylated antibody clone was used as the detector at a concentration of 0.3 µg ml^−1^. The SBG revelation enzyme concentration was 150 pM. Recombinant IFN-α2c (eBioscience) was used as the calibrator. For the IFN-γ assay, the MD-1 antibody clone (BioLegend) was used as a capture antibody after coating on paramagnetic beads (0.3 mg ml^−1^), and the 25718 antibody clone (R&D Systems) was biotinylated (biotin:antibody ratio of 40/1) and used as the detector antibody at a concentration of 0.3 µg ml^−1^. The SBG revelation enzyme concentration was 150pM. Recombinant protein (PBL Assay Science) was used to quantify IFN-γ concentrations. For the IL-17A assay, the BL23 antibody clone (BioLegend) was used as a capture antibody after coating on paramagnetic beads (0.3 mg ml^−1^), and the MT504 antibody clone (MabTech), already biotinylated, was used as the detector antibody at a concentration of 0.3 µg ml^−1^. The SBG revelation enzyme concentration was 150 pM. For the IFN-β assay, the 710322-9 IgG1, kappa, mouse monoclonal antibody (PBL Assay Science) was used as a capture antibody after coating paramagnetic beads (0.3 mg ml^−1^), the 710323-9 IgG1, kappa, mouse monoclonal antibody (PBL Assay Science) was biotinylated (biotin:antibody ratio of 40/1) and used as the detector antibody, and recombinant protein (PBL Assay Science) were used to quantify IFN-β concentrations. For the IFN-λ3 assay, the MMHL-3 IgG1 kappa mouse monoclonal antibody (PBL Assay Science) was used as a capture antibody after coating paramagnetic beads (0.3 mg ml^−1^), the 567107 R IgG2a mouse monoclonal antibody (R&D systems) was biotinylated (biotin:antibody ratio of 60/1) and used as the detector antibody, and recombinant protein (PBL Assay Science) were used to quantify IFN-λ3 concentrations. The limits of detection of these assays were: 0.6 pg ml^−1^ for IFN-β, 0.6 pg ml^−1^ for IFN-λ3, 2 fg ml^−1^ for IFN-α, 7 fg ml^−1^ for IFN-γ and 3 pg ml^−1^ for IL-17A, including the dilution factor. An additional 38 cytokines and chemokines were measured in plasma and nasal supernatants with a commercial Luminex multi-analyte assay (Biotechne, R&D systems). Data were acquired on a Bio-Plex 200 System (Bio-Rad) and analyzed with Bio-Plex Manager v5 (Bio-Rad). Nasopharyngeal mucin levels were analyzed using a MUC5AC ELISA Kit (NBP2-76703, Novus Biologicals).

### Quantification of nasopharyngeal viral load

Nasopharyngeal viral loads were determined using RdRp-IP4 RT–qPCR designed at the Institut Pasteur (National Reference Center for Respiratory Viruses) to target a section of the *RdRP* gene based on the first sequences of SARS-CoV-2 made available on the Global Initiative on Sharing All Influenza Data database on 11 Jan 2020 (ref. ^51^). Primer and probe sequences were: nCoV_IP4-14059Fw GGTAACTGGTATGATTTCG; nCoV_IP4-14146Rv CTGGTCAAGGTTAATATAGG; nCoV_IP4-14084Probe^+^ TCATACAAACCACGCCAGG [5′]Fam [3′]BHQ-1. All positive samples were quantified using a standard curve and expressed as the number of RNA copies per ml. Mucin levels were analyzed using a MUC5AC ELISA Kit (NBP2-76703, Novus Biologicals). Data were collected with the Multiskan Spectrum (Thermo Fisher Scientific).

### Quantification of plasma viral load

SARS-CoV-2 viremia was quantified in each patient blood sample using the Naica droplet-based digital PCR machine (Stilla) with COVID-19 Multiplex Crystal digital PCR detection kit (Apexbio) as previously described^52^. Plasma viral RNA was extracted using QIAamp Viral RNA Mini Kit, following the manufacturer’s guidelines. Results were automatically analyzed using ‘Crystal Reader’ and ‘Crystal Miner’ software and SARS-CoV-2 viral concentrations (cp ml^−1^) were finally calculated considering the extracted volume of plasma (140 µl).

### Plasma pseudotype neutralization assay

293T cells stably expressing ACE2 (293T-ACE2) were made by lentiviral transduction and selection with puromycin (1 µg ml^−1^). To perform the assay, 2 × 10^4^ cells were detached with PBS-EDTA and seeded in flat-bottom 96-well plates. S-pseudotypes were incubated with the sera to be tested (at 1:100 dilution, unless otherwise specified) in culture medium, incubated for 15 min at room temperature and added on transduced cells. After 48 h, Bright-Glo Luciferase Assay System was added to the wells and the luciferase signal was measured with EnSpire Multimode Plate Reader (PerkinElmer). The percentage of neutralization was calculated as follows: 100 × (1 **−** mean (luciferase signal in sample duplicate)/mean (luciferase signal in virus alone)). S-pseudotypes incubated without serum and medium alone were used as positive and negative controls, respectively.

### Nasopharyngeal S-Fuse neutralization assay

The S-Fuse assay^18,53^ was used to assess nasopharyngeal SARS-CoV-2-neutralizing antibodies. U2OS-ACE2 GFP1-10 or GFP11 cells, also termed S-Fuse cells, become positive for GFP when they are productively infected by SARS-CoV-2. The Wuhan Hu-1 SARS-CoV-2 virus strain (wild type) was incubated with control monoclonal antibodies or nasopharyngeal samples before adding to S-Fuse cells. At 18 h later, cells were fixed with 2% paraformaldehyde, washed and stained with Hoechst (dilution 1:1,000; Invitrogen), and GFP^+^ cells were imaged using an Opera Phenix high-content confocal microscope (PerkinElmer). The percentage of neutralization was calculated using the number of syncytia as the value with the following formula: 100 × (1 – (value with serum – value in ‘non-infected’)/(value in ‘no serum’ – value in ‘non-infected’)). Neutralizing activity of each sample was expressed as ED_50_ values (in μg ml^−1^ for monoclonal antibodies and in dilution values for nasopharyngeal samples), which were calculated with a reconstructed curve using the percentage of neutralization at the different concentrations.

### Bacterial DNA isolation and 16S rRNA sequencing

We extracted total genomic DNA from swab samples, using the NucleoSpin 96 Genomic DNA kit (Macherey-Nagel). Negative control samples included buffers only. Briefly, the pellets were incubated with Ready-Lyse Lysozyme Solution (250 U μl^−1^; Epicentre) for 30 min at 37 °C followed by Proteinase K digestion buffer at 55 °C overnight. Carrier (20 μg glycogen) was added and DNA extraction was performed according to the manufacturers’ instructions. DNA was eluted in 25 μl of solution and immediately frozen at −80 °C. The concentration of extracted DNA was determined using TECAN (QuantiFluor ONE dsDNA System, Promega), and DNA integrity and size were also confirmed with the Agilent 2100 Bioanalyzer. The V3–V4 region of bacterial 16S rRNA was amplified using V3-340F (CCTACGGRAGGCAGCAG) and V4-805R (GGACTACHVGGGTWTCTAAT) primers^54,55^. The primers have a primer linker, primer pad and unique 8-mer Golay barcode, which was used to tag PCR products from respective samples and negative control. PCR reactions consisted of 18 μl of AccuPrime Pfx Super Mix (12344-040; Invitrogen), 0.5 μl of each primer and 1 μl of DNA (10 ng). PCR was carried out as follows: 95 °C for 2 min, 30 cycles of 95 °C for 20 s, 55 °C for 15 s and 72 °C for 1 min, and a final extension step at 72 °C for 10 min on a Bio-Rad thermocycler. PCR products were cleaned using Nucleo Mag magnetic purification beads (Macherey-Nagel) following the protocol, quantified with the Quanti Fluor ONE dsDNA kit (Promega), and pooled in equal amounts of each PCR product. Library pools were loaded at 12 pM with a 15% PhiX spike for diversity and sequencing control, onto a v3 300-bp paired-end reads cartridge for sequencing on the Illumina MiSeq next-generation sequencing platform. The raw sequence data for each sample were deposited in the NCBI Sequence Read Archive (SUB9287653/BioProject ID: PRJNA714242).

### Sequence processing and statistical analysis

After removing reads containing incorrect primer or barcode sequences and sequences with more than one ambiguous base, we recovered a total of 8,075,384 reads (192,271 reads on average) from 42 samples. The bioinformatics analysis was performed into OTUs^27^ or into ASVs^28^. Briefly, amplicons were clustered into OTUs with VSEARCH (v1.4) or ASV and aligned against the SILVA database. The input amplicons were then mapped against the OTU/ASV set to get an OTU/AVS-abundance table containing the number of reads associated with each OTU/ASV. The normalization, statistical analyses and multiple visualization were performed with SHAMAN (SHiny application for Metagenomic Analysis (http://shaman.c3bi.pasteur.fr/) based on R software.

### Bacterial load quantification

Universal 16S rRNA primers were used to quantify total bacterial load (16 S_F: 5′-ATTACCGCGGCTGCTGG-3′ and 16S and 16S_R: 5′-ATTACCGCGGCTGCTGG-3′). PCR reactions consisted of 10 μl SYBR Green PCR master mix (Roche), 1 μl (10 nM) of each primer and 200 ng of template cDNA in 20 μl of reaction carried out on an ABI StepOnePlus Sequence Detection System (Applied Biosystems). Thermocycling reactions consisted of 1 min at 95 °C followed by 40 cycles of 15 s at 95 °C, 15 s at 56 °C and 45 s at 72 °C.

### Statistical analysis

GraphPad Prism v9 was used for statistical analysis. Cytokines were filtered first on variance (*σ* > 2) to remove analytes where the majority of values were at or close to the limit of detection. *P* values were determined by a Kruskal–Wallis test, followed by Dunn’s post hoc test for multiple comparisons with median values reported, or all datasets were compared using nonparametric two-tailed Mann–Whitney tests. *P < 0.05; **P < 0.01; ***P < 0.001. Correlations between the different assays were calculated using the Spearman test. Heat maps were generated with Qlucore OMICS explore version 3.5. Correlation matrices were built using the Spearman correlation, and computed in R (v4.0.3). Correlation plots were generated with the R package ‘corrplot’ (v0.84). The MDS plots were derived from the correlation matrices by defining a similarity metric equal to ‘1 − *R*_s_(a,b)’, where *R*_s_(a,b) is the spearman correlation between factor a and b. MDS computation was performed with the ‘cmdscale’ function from the ‘stats’ package (v4.0.3). Plots were made using the ggplot2 package (v3.3.2).

### Reporting Summary

Further information on research design is available in the Nature Research Reporting Summary linked to this article.

## Online content

Any methods, additional references, Nature Research reporting summaries, source data, extended data, supplementary information, acknowledgements, peer review information; details of author contributions and competing interests; and statements of data and code availability are available at 10.1038/s41590-021-01028-7.

## Supplementary information

Reporting Summary

Supplementary Data 1 Statistical source data.

Supplementary Table 1 Patient information.

## Data Availability

The datasets generated during and/or analyzed in the current study are available from the corresponding authors upon request.

## Code availability

The code used in the current study is available from the corresponding authors upon request.
