# Supplementary material for: Distinct systemic and mucosal immune responses during acute SARS-CoV-2 infection
Source: Nat Immunol. 2021 Sep 1;22(11):1428–39. doi: 10.1038/s41590-021-01028-7 (PMC8553615; doi:10.1038/s41590-021-01028-7)
Supplement: Supplementary file 1 — Reporting Summary [file 41590_2021_1028_MOESM1_ESM.pdf]

nature research

Corresponding author(s): James Di Santo

Last updated by author(s): Aug 3, 2021

Reporting Summary

Nature Research wishes to improve the reproducibility of the work that we publish. This form provides structure for consistency and transparency in reporting. For further information on Nature Research policies, see our Editorial Policies and the Editorial Policy Checklist.

Statistics

For all statistical analyses, confirm that the following items are present in the figure legend, table legend, main text, or Methods section.

n/a	Confirmed	
☐	☒	The exact sample size (*n*) for each experimental group/condition, given as a discrete number and unit of measurement
☐	☒	A statement on whether measurements were taken from distinct samples or whether the same sample was measured repeatedly
☐	☒	The statistical test(s) used AND whether they are one- or two-sided<br>*Only common tests should be described solely by name; describe more complex techniques in the Methods section.*
☐	☒	A description of all covariates tested
☐	☒	A description of any assumptions or corrections, such as tests of normality and adjustment for multiple comparisons
☐	☒	A full description of the statistical parameters including central tendency (e.g. means) or other basic estimates (e.g. regression coefficient) AND variation (e.g. standard deviation) or associated estimates of uncertainty (e.g. confidence intervals)
☐	☒	For null hypothesis testing, the test statistic (e.g. *F*, *t*, *r*) with confidence intervals, effect sizes, degrees of freedom and *P* value noted<br>*Give P values as exact values whenever suitable.*
☒	☐	For Bayesian analysis, information on the choice of priors and Markov chain Monte Carlo settings
☒	☐	For hierarchical and complex designs, identification of the appropriate level for tests and full reporting of outcomes
☐	☒	Estimates of effect sizes (e.g. Cohen's *d*, Pearson's *r*), indicating how they were calculated

Our web collection on statistics for biologists contains articles on many of the points above.

Software and code

Policy information about availability of computer code

Data collection	AttuneTM NxT Flow cytometer (Thermo Fisher); Bio-Plex 200 System (Bio-Rad); Simoa HD-1 analyzer (Quanterix); Multiskan® Spectrum (Thermo Fisher Scientific)
Data analysis	Qlucore Omics Explorer V3.6; GraphPad Prism v9; R v4 package `corrplot`(v0.84); FlowJo v10; Bio-Plex Manager v5; AttuneTM NxT Software 3.2.1243.0;

For manuscripts utilizing custom algorithms or software that are central to the research but not yet described in published literature, software must be made available to editors and reviewers. We strongly encourage code deposition in a community repository (e.g. GitHub). See the Nature Research guidelines for submitting code & software for further information.

Data

Policy information about availability of data

All manuscripts must include a data availability statement. This statement should provide the following information, where applicable:

- Accession codes, unique identifiers, or web links for publicly available datasets
- A list of figures that have associated raw data
- A description of any restrictions on data availability

The 16S data will be available online soon. For the other data the availability can be by request to the authors

Field-specific reporting

Please select the one below that is the best fit for your research. If you are not sure, read the appropriate sections before making your selection.

☒ Life sciences ☐ Behavioural & social sciences ☐ Ecological, evolutionary & environmental sciences

For a reference copy of the document with all sections, see nature.com/documents/nr-reporting-summary-flat.pdf

Life sciences study design

All studies must disclose on these points even when the disclosure is negative.

Sample size	No sample size was chosen. Inclusion criteria for COVID-19 inpatients were: age between 18 and 80 years old, diagnosis of COVID-19 according to WHO interim guidance, and positive SARS-CoV-2 RT-PCR testing on a respiratory sample (nasopharyngeal swab or invasive respiratory sample). Inpatients with pre-existing unstable chronic disorders (such as uncontrolled diabetes mellitus, severe obesity defined as body mass index greater than 30, unstable chronic respiratory disease or chronic heart disease) and with bacterial co-infection were excluded. Since median duration from onset of symptoms to respiratory failure was previously shown to be 9.5 (interquartile range, 7.0-12.5) days 53, we analyzed immune responses between 8 to 12 days after onset of first symptoms for all patients and before the initiation of any antiviral or anti-inflammatory treatment. Healthy controls were asymptomatic adults, matched with cases on age (+/- 5 years), with a negative SARS-CoV-2 RT-PCR testing at time of inclusion. We didn't use statistics to calculate the sample size because we took all samples/patients available (after inclusion criteria were respected and consent were given)
Data exclusions	No data were excluded
Replication	All attempt of replication were successfull. We perform paired analysis of the nasopharyngeal and plasma samples of other cohorts of SARS-CoV-2 infected patients and we replicated the distinct compartmentalization of anti-Spike antibody responses, cytokine production and shifts in the nasopharyngeal microbiome.
Randomization	COVID-19 patients and healthy controls' samples were recieved in a randomized order by the clinicians, and were treated this way. Allocation could not be randomized as we compare Healthy controls versus COVID-19 patients samples. Covariates were controled between the two groups by matching the age and sex. Both groups were selected as they didn't present any comorbidities. Healthy donors : 71% of male; mean age of 61,2 years old (min : 28,3yo; max: 70,5yo) and COVID-19 patients : 76% of male; mean age of 53,1 years old (min : 25,7yo; max: 79,2yo).
Blinding	Investigators were blinded to group allocation during acquisition and analysis of data.

Reporting for specific materials, systems and methods

We require information from authors about some types of materials, experimental systems and methods used in many studies. Here, indicate whether each material, system or method listed is relevant to your study. If you are not sure if a list item applies to your research, read the appropriate section before selecting a response.

Materials & experimental systems

n/a	Involved in the study	
☐	☒	Antibodies
☐	☒	Eukaryotic cell lines
☒	☐	Palaeontology and archaeology
☒	☐	Animals and other organisms
☐	☒	Human research participants
☒	☐	Clinical data
☒	☐	Dual use research of concern

Methods

n/a	Involved in the study	
☒	☐	ChIP-seq
☐	☒	Flow cytometry
☒	☐	MRI-based neuroimaging

Antibodies

Antibodies used	mAb48 and mAbC1-169 used in S-Flow/S-Fuse are human anti-S monoclonal antibodies isolated and produced by Hugo Mouquet Lab (Institut Pasteur). S-Flow cells were stained with anti-IgG Alexa Fluor 647 (Cat # A-21445, Thermo Fisher Scientific) or anti-IgA Alexa Fluor 647 (Cat # 109-605-011, Jackson ImmunoResearch).<br>For Simoa the following antibodies were used: Anti-IFN-α human antibody BMS216C clone (eBioscience)<br>Anti-IFN-α human antibody BMS216BK clone (eBioscience); Anti-IFN-γ human antibody MD-1 clone (BioLegend); Anti-IFN-γ human antibody 25718 clone (R&D Systems); Anti-IL-17A human antibody BL23 clone (BioLegend); Anti-IL-17A human antibody MT504 clone (MabTech); Anti-IFN-β human antibody 710322-9 clone (PBL Assay Science); Anti-IFN-β human antibody 710323-9 clone (PBL Assay Science); Anti-IFNL3 human antibody MMHL-3 clone (PBL Assay Science); Anti-IFNL3 human antibody 567107R clone (R&D systems)
Validation	mAb48 (IgG) and mAbC1-169 (IgA) human monoclonal antibodies were validated using ELISA binding assays (against the trimeric S, RBD, and S2 proteins of Wuhan Hu-1 SARS-CoV-2 virus strain) by the team of Hugo Mouquet. Validation of anti-IgG (A-21445 clone)

and anti-IgA (109-605-011 clone) human secondary antibodies is available from the manufacturer's website. S-Flow characteristics including sensitivity and specificity were previously described (Grzelak, L. et al. Sci Transl Med (2020))

The validation of antibodies used on Simoa is available from the manufacturer's website and were previously described: Hadjadj et al., Impaired type I interferon activity and inflammatory responses in severe COVID-19 patients. Science 369 (2020).

Validation of antibodies available from the manufacturer's website:
Anti-IgA 109-605-011 clone (Jackson ImmunoResearch) - "Based on immunoelectrophoresis and/or ELISA, the antibody reacts with the heavy chain of human IgA but not with human IgG or IgM. No antibody was detected against non-immunoglobulin serum proteins. The antibody may cross-react with IgA from other species."
Anti-IgG A-21445 clone (Thermo Fisher Scientific) - "to minimize cross-reactivity, these goat anti-human IgG (H+L) whole secondary antibodies have been affinity purified and cross- adsorbed against mouse, rabbit, and bovine serum prior to conjugation. For the fluorophore-labeled antibodies a final concentration of 1-10 µg/mL should be satisfactory for most immunohistochemistry and flow cytometry applications"
Anti-IFN-α human antibody BMS216C clone (eBioscience) - "specifically reacts with human recombinant and natural interferon alpha and can be used as primary antibody in ELISA"
Anti-IFN-α human antibody BMS216BK clone (eBioscience) - "specifically reacts with human recombinant and natural interferon alpha and can be used as capture antibody in ELISA"
Anti-IFN-γ human antibody MD-1 clone (BioLegend) - "Each lot of this antibody is quality control tested by ELISA assay. For ELISA Capture applications, the antibody should be titrated between 0.25 - 2 µg/ml to determine optimal condition"
Anti-IFN-γ human antibody 25718 clone (R&D Systems) - "Detects human IFN-gamma in direct ELISAs. In direct ELISAs, no cross-reactivity with recombinant mouse IFN-gamma, recombinant rat IFN-gamma, or recombinant porcine IFN-gamma is observed."
Anti-IL-17A human antibody BL23 clone (BioLegend) - "Each lot of this antibody is quality control tested by ELISA assay. For use as an ELISA capture antibody, a concentration range of 0.25 - 1.0 µg/ml is recommended."
Anti-IL-17A human antibody MT504 clone (MabTech) - "This monoclonal antibody is recommended for neutralization of human IL-17A bioactivity. The antibody cross-reacts with IL-17A from non-human primates. Cross-reactivity has been tested in ELISA and/or ELISpot."
Anti-IFN-β human antibody 710322-9 clone (PBL Assay Science) - "Binds to human IFN-beta with high affinity"
Anti-IFN-β human antibody 710323-9 clone (PBL Assay Science) - "Binds to human IFN-beta with high affinity"
Anti-IFNL3 human antibody MMHL-3 clone (PBL Assay Science) - "Mouse monoclonal antibody against Human IL-28B/IFNL3. Less than 0.1% cross-reactivity is observed with human IFNL1 and human IFNL2"
Anti-IFNL3 human antibody 567107R clone (R&D systems) - "Detects human IL-28B/IFN- lambda 3 in ELISAs."

Eukaryotic cell lines

Policy information about cell lines

Cell line source(s)	Human embryonic kidney (HEK) 293T (referred as 293T) cells (ATCC CLR-3216) and human bone osteosarcoma epithelial (referred as U2OS) cells (ATCCr HTB-96TM) were from the American Type Culture Collection (ATCC) and were grown in complete DMEM medium (10% Fetal Calf Serum, 1% Penicillin/ streptomycin).
Authentication	Cell lines used were not authenticated
Mycoplasma contamination	All cells are negative for mycoplasma contamination. Cells were tested for absence of mycoplasma contamination using Mycoalert TM Mycoplasma Detection Kit (Lonza). Tests were performed on a month basis.
Commonly misidentified lines (See ICLAC register)	None

Human research participants

Policy information about studies involving human research participants

Population characteristics	Inclusion criteria for COVID-19 inpatients were: age between 18 and 80 years old, diagnosis of COVID-19 according to WHO interim guidance, and positive SARS-CoV-2 RT-PCR testing on a respiratory sample (nasopharyngeal swab or invasive respiratory sample). Inpatients with pre-existing unstable chronic disorders (such as uncontrolled diabetes mellitus, severe obesity defined as body mass index greater than 30, unstable chronic respiratory disease or chronic heart disease) and with bacterial co-infection were excluded. Since median duration from onset of symptoms to respiratory failure was previously shown to be 9.5 (interquartile range, 7.0-12.5) days 53, we analyzed immune responses between 8 to 12 days after onset of first symptoms for all patients and before the initiation of any antiviral or anti-inflammatory treatment. Healthy controls were asymptomatic adults, matched with cases on age (+/- 5 years), with a negative SARS-CoV-2 RT-PCR testing at time of inclusion. Healthy donors : 71% of male; mean age of 61,2 years old (min : 28,3yo; max: 70,5yo) and COVID-19 patients : 76% of male; mean age of 53,1 years old (min : 25,7yo; max: 79,2yo).
Recruitment	Inclusion criteria for COVID-19 inpatients were: age between 18 and 80 years old, diagnosis of COVID-19 according to WHO interim guidance, and positive SARS-CoV-2 RT-PCR testing on a respiratory sample (nasopharyngeal swab or invasive respiratory sample). Inpatients with pre-existing unstable chronic disorders (such as uncontrolled diabetes mellitus, severe obesity defined as body mass index greater than 30, unstable chronic respiratory disease or chronic heart disease) and with bacterial co-infection were excluded. Since median duration from onset of symptoms to respiratory failure was previously shown to be 9.5 (interquartile range, 7.0-12.5) days 53, we analyzed immune responses between 8 to 12 days after onset of first symptoms for all patients and before the initiation of any antiviral or anti-inflammatory treatment. Healthy controls were asymptomatic adults, matched with cases on age (+/- 5 years), with a negative SARS-CoV-2 RT-PCR testing at time of inclusion. Every patient that fit the inclusion criteria during the time of recruitment was offered to participate in the study. After the consent was obtained, the patients were included. No biais selection needs to be delcare.

Ethics oversight	The study conforms to the principles outlined in the Declaration of Helsinki, and received approval by the appropriate Institutional Review Board (Cochin-Port Royal Hospital, Paris, France; number AAA-2020-08018).

Note that full information on the approval of the study protocol must also be provided in the manuscript.

Flow Cytometry

Plots

Confirm that:

☒ The axis labels state the marker and fluorochrome used (e.g. CD4-FITC).

☒ The axis scales are clearly visible. Include numbers along axes only for bottom left plot of group (a 'group' is an analysis of identical markers).

☒ All plots are contour plots with outliers or pseudocolor plots.

☒ A numerical value for number of cells or percentage (with statistics) is provided.

Methodology

Sample preparation	Human embryonic kidney (HEK) 293T (referred as 293T) cells were from the American Type Culture Collection (ATCC) (ATCC CRL-3216) and tested negative for mycoplasma. Cells were split every 2 to 3 days using Dulbecco's modified Eagle's medium (DMEM) supplemented with 10% fetal calf serum and 1% penicillin-streptomycin (complete medium). A codon-optimized version of the SARS-Cov-2 S gene (GenBank: QHD43416.1) (1) was transferred into the phCMV backbone (GenBank: AJ318514) by replacing the VSV-G gene. 293T cells were transfected with S or a control plasmid using Lipofectamine 2000 (Life Technologies). One day after, transfected cells were detached using PBS-EDTA and transferred into U-bottom 96-well plates (50,000 cells per well). Cells were incubated at 4°C for 30 min with plasma (1:300 dilution) or nasal swab (1:5 dilution) in PBS containing 0.5% BSA and 2 mM EDTA, washed with PBS, and stained using either anti-IgG Alexa Fluor 647 (Thermo Fisher Scientific) or anti-IgA Alexa Fluor 647 (Jackson ImmunoResearch). Cells were washed with PBS and fixed for 10 min using 4% paraformaldehyde (PFA). Specific binding was calculated with the following formula: 100 × (% binding on 293T-S – binding on control cells)/(100 – binding on control cells).
Instrument	Data were acquired on an Attune NxT instrument (Life Technologies).
Software	FlowJo 10.7.1<br>AttuneTM NxT Software 3.2.1243.0
Cell population abundance	Singlets were around 80% of the events, around 60-70% of the singlets were selected in the "Cells" gate
Gating strategy	Singlets were first selected as a diagonal on FSC-H against FSC-A. Cells were then gated in SSC-A against FSC-A. The Ig gate was put according to the control cells staining for each sample as the control cells were set as negative.

☒ Tick this box to confirm that a figure exemplifying the gating strategy is provided in the Supplementary Information.
